# Quinex: Quantitative information extraction from text using open and lightweight LLMs

**DOI:** 10.1016/j.xinn.2026.101391

**Published:** 2026-04-15

**Authors:** Jan Göpfert, Patrick Kuckertz, Gian Müller, Luna Lütz, Celine Körner, Hang Khuat, Detlef Stolten, Jann Michael Weinand

**Affiliations:** 1 Forschungszentrum Jülich GmbH, Institute of Climate and Energy Systems – Jülich Systems Analysis, 52425 Jülich, Germany; 2 RWTH Aachen University, Chair for Fuel Cells, Faculty of Mechanical Engineering, 52062 Aachen, Germany

**Keywords:** quantitative information extraction, scientific literature mining, small language models, automated literature review, structured data extraction, scientific NLP

## Abstract

Extracting quantitative data from the growing body of scientific literature is a challenge central to modern research across disciplines. While recent advances in large language models have significantly facilitated automation of this traditionally time-consuming task, their computational demands limit scalability and accessibility. Smaller specialized systems offer reduced computational requirements but sacrifice accuracy, domain generalization, or the extraction of contextual details. To address these gaps, we present Quinex, a domain-agnostic framework for quantitative information extraction based on comparably small language models. Quinex identifies quantities and their associated entities, properties, and qualifiers using a multi-turn question-answering approach. By addressing the data bottleneck, considering implicit properties, and optimizing extraction order and question templates, Quinex achieves state-of-the-art F1 scores of over 98% for quantities, 82% for entities, and 87% for properties across diverse scientific genres. It goes beyond identification by normalizing units, aligning them with a unit ontology, and detailing critical qualifiers such as references, spatiotemporal scopes, and determination methods. By reducing the effort required to extract structured quantitative data from texts, Quinex enables transformative applications, including automated literature reviews, quantitative search, and trend monitoring, and sets a new benchmark for scalable, accurate, and domain-agnostic quantitative information extraction.

## Public summary

• Quinex, an efficient and domain-agnostic tool for extracting quantitative data from text, is presented.
• Quinex facilitates large-scale literature analyses and trend monitoring for a broad audience.
• Small, fine-tuned language models achieve state-of-the-art results for lightweight systems.
• To achieve this, significantly larger training datasets spanning diverse research fields were created.
• Qualitative improvements include consideration of implicit properties and detailed, critical qualifiers.

## Graphical abstract

**Figure:**
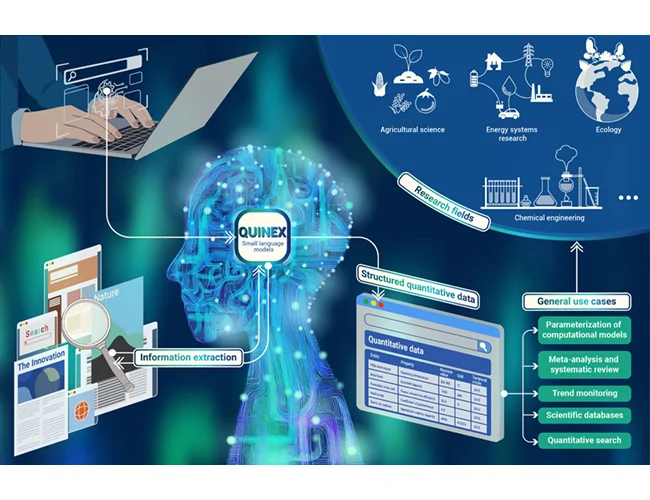

## Introduction

Many research fields depend on collecting quantitative information from literature, though this process can be challenging. In microbiology, for example, when compiling data on bacteria and archaea, including experimental results, it is difficult to extract from primary sources.^1^ In materials science, substantial efforts have been made to manually gather experimental data on perovskite solar cells.^2^ Similarly, maintaining up-to-date life cycle assessment databases requires more researchers than are typically available.^3^ In climate and energy research, the robustness of techno-economic assumptions in integrated assessment and energy system models relies on extensive analysis of the scientific literature.^4^^,^^5^

Untangling large amounts of information from unstructured documents, such as scientific articles or patents, is a tedious task; hence, systems for automating the extraction of quantities and their measurement context have been developed.^6^ Early, predominantly rule-based systems allowed for high precision in narrowly defined applications.^6^ Before the dominance of transformer-based models^7^ and, in particular, BERT-based models,^8^ machine learning approaches often relied on conditional random field models^9^ and bidirectional long short-term memory models.^10^ Among others, the extraction of quantities and what they measure has been framed as sequence-labeling tasks,^11^^,^^12^^,^^13^^,^^14^ combinations of sequence-labeling and question-answering (QA) tasks,^15^^,^^16^ relation-extraction tasks,^17^^,^^18^^,^^19^ template-filling tasks,^20^^,^^21^^,^^22^^,^^23^ as well as event-extraction tasks^24^, and has been approached from the perspective of open information extraction.^25^ At the time of this work, the capabilities of recent large autoregressive language models (e.g., zero- and few-shot learning with GPT and Llama models^26^^,^^27^) and the prompting strategies they enable remain largely unexplored. Fine-tuning-based approaches for extracting measurement contexts have been limited by the amount of training data available.^6^

**Figure 1:**
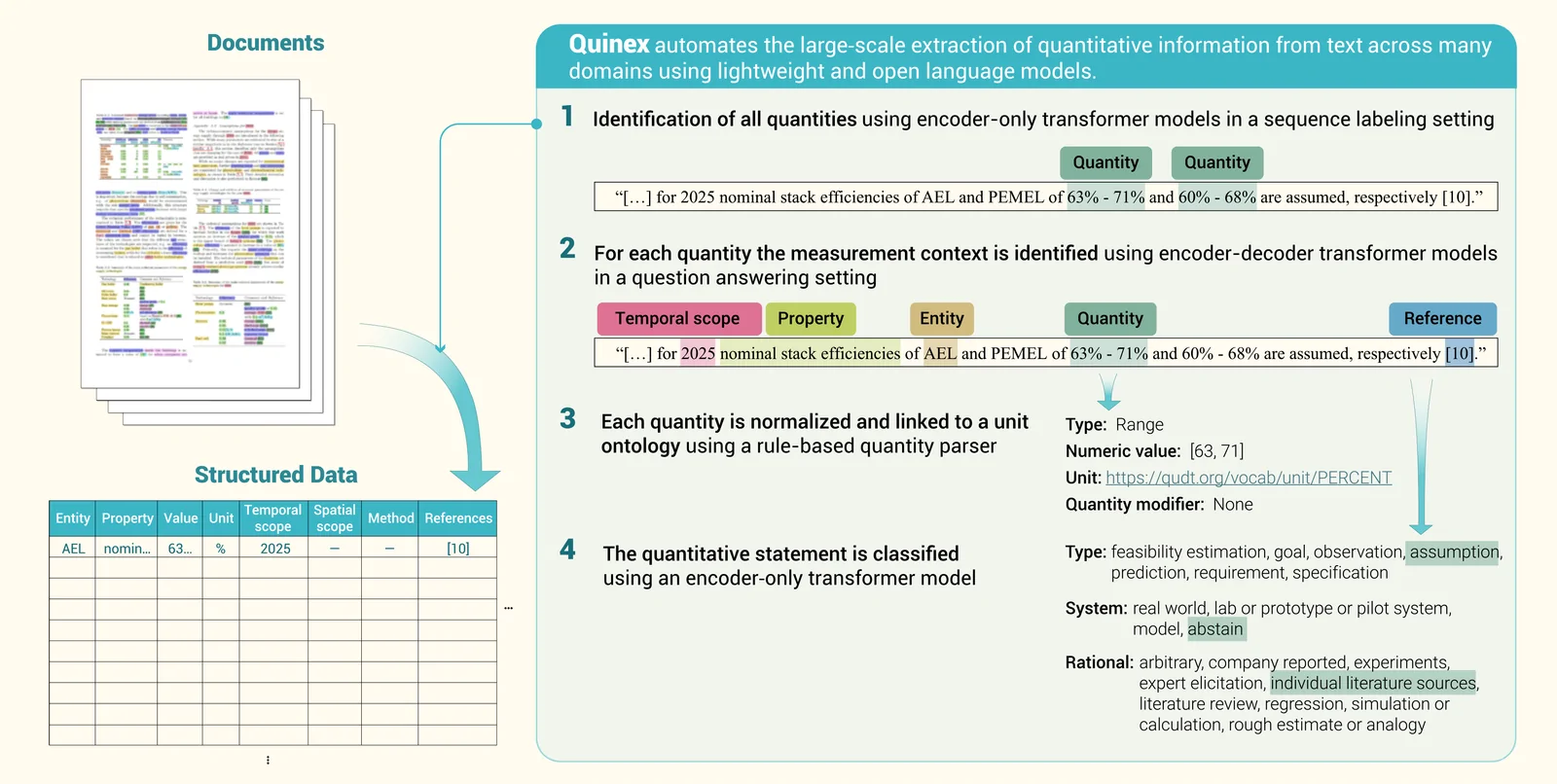
Schematic illustration of Quinex’s approach to quantitative information extraction Quinex automates the domain-agnostic, large-scale extraction of quantitative information from text using lightweight and open language models in a pipeline approach, where all quantities are identified before their measurement context is extracted. Quantities are normalized, and the quantitative statement is classified.

In this work, we detail a system for the extraction of quantities and their contexts from text (see Figure 1): Quinex identifies quantity spans—that is, numeric values, along with their units of measurement and modifiers, if applicable. A quantity span can be a single quantity or composed of multiple quantities, e.g., intervals, lists, or multidimensional quantities. Quantities are normalized and units mapped to theQuantities, Units, Dimensions, and Types (QUDT) ontology,^28^ e.g., “less than four TWh” is normalized to (“<”, “4”, “qudt:TeraW-HR”). For each quantity, Quinex extracts the measured entity and property. A notable difference to many other systems is that both explicitly and implicitly given measured properties are extracted. This is important, as properties are often given implicitly, e.g., rated power is not mentioned in “… the 6 MW turbine …” As the value of a measurement only holds true under specific constraints, we also extract the qualifiers for each quantitative statement, i.e., the temporal scope, spatial scope, references, determination method, and other qualifiers. Finally, the quantitative statements are classified according to whether they describe a model or a real system, an assumption or a specification, and so on.

By addressing the data bottleneck, adapting the task design, and performing parameter variations that reveal advantageous approaches, we achieve strong results with open and lightweight large language models (LLMs). This enables quantitative information extraction at scale for a large audience and will aid research in many fields by facilitating literature searches and the enrichment of scientific databases.

## Materials and methods

In this work, quantitative information extraction is divided into multiple subtasks, which are solved using specialized components. Below, we first describe the framework’s architecture, its components, and the chosen approaches. Secondly, as the core components are based on machine learning, we outline the creation of datasets for training and evaluation purposes. Finally, we outline the framework's implementation and present the experimental setup for its empirical assessment.

### Framework architecture

Quantities are easy to spot and almost always stated explicitly, which makes them ideal anchors for all further operations. Hence, we follow a cascaded approach, in which we identify quantities in a first stage and extract their measurement context in a second stage (see Figure 1). This was a common design pattern among the systems we reviewed earlier.^6^ After quantity spans have been identified, they are normalized, the measurement context is extracted, and the quantitative statement is classified. The measurement context is extracted in three stages. In the first two stages, the measured entity and property are identified, and in the third stage, the qualifiers are identified. Predictions from previous stages are incorporated into the input for subsequent stages. Whether to identify entities or properties first is examined empirically (see the results section). The qualifiers are extracted last because they often refer to the entire quantitative claim and therefore benefit from stating the claim in full, which presupposes that the quantity, property, and entity have already been identified. To reduce latency, measurement context extraction and statement-type classification are performed in parallel. Similarly, all qualifiers are extracted simultaneously based on the assumption that predictions for one qualifier do not significantly aid the identification of another. In the following sections, the approaches for each individual component are outlined.

#### Quantity span identification

Since quantities are almost always given explicitly, we cast quantity span identification as a sequence-labeling problem. Each token is classified as being inside, outside, or at the beginning of a quantity span (IOB tagging).^29^ For this, we use small, encoder-only transformer models because they perform well, have bidirectional representations, and require little computational resources. Although today’s autoregressive LLMs provide strong zero- and few-shot capabilities, in tasks traditionally approached by sequence labeling, such as named entity recognition (NER), they are often outperformed by comparable small, fine-tuned encoder-only models.^30^^,^^31^^,^^32^^,^^33^

#### Quantity span normalization

Quantity span normalization is performed using a rule-based parser that dissects quantity spans into numeric values, units, modifiers, and uncertainty expressions. The parser determines the type of quantity span (single quantity, range, list, ratio, multidimensional, or unknown) and normalizes values, units, modifiers, and uncertainty expressions. Units are normalized by linking them to their corresponding QUDT unit class and determining the exponent. The aim is not to divide the compound units into their smallest parts but to return as few parts as possible; ideally, a single QUDT class is equivalent to the given unit. The algorithms are described in more detail in the supplemental material and methods S1.2 and are published open-source.

#### Measurement context extraction

Measurement context extraction is framed as multi-turn QA. Traditionally, named entity and relation extraction is performed sequentially. First, all named entity spans are identified, and subsequently, the relations between entity pairs are determined. When framing entity and relation extraction as multi-turn QA, entities and relations are jointly extracted, shortening the error cascade.^34^ Furthermore, by identifying entities with relation-specific questions, valuable information can be encoded in the questions,^35^ and relational triples that overlap with each other do not pose a problem.^34^ As explained earlier, quantity spans are ideal anchors for relation-specific tagging. Both QA and relation-specific tagging have been successful approaches in the MeasEval challenge.^36^

For each quantity, the related measured entity, property, and qualifiers are extracted using templated questions. Already predicted answers are inserted in the slots of the question templates. The property is identified first, and the result is inserted in the entity question template, or vice versa. The identified entity and property are then inserted into the templates for qualifier extraction. If no related property or entity can be identified, fallback questions without the respective slots are used. A detailed flow diagram of Quinex is given in Figure S1. Consider the sentence, “The nominal power and rotor diameter of the Enercon E-175 EP5 are 6 MW and 175 m, respectively.” The quantity model identifies two quantities in this sentence, namely “6 MW” and “175 m.” For both, the related entity and property are identified by successively asking for them, i.e., we prefix the sentence with a corresponding question and predict the answer using a language model fine-tuned on QA. In this example, we ask for the property that is quantified by 6 MW by inserting the quantity span into the following question template:

“Which property or quality is characterized by {*quantity*_*span*}?”

To get the measured entity, we further ask, inserting the quantity span and predicted property,

“Which entity’s {*property*_*span*} is characterized by {*quantity*_*span*}?”

If the model abstains from answering the property question, because the property is not clear from the given context, we use a fallback question instead:

“Which entity does {*quantity*_*span*} characterize?”

To get further context, such as the temporal scope, we ask further questions. If we have non-empty predictions for both the property and entity, we ask,

“For which point in time is the statement true that {*property*_*span*} of {*entity*_*span*} is {*quantity*_*span*}?”

If one is missing, we use the fallback question instead:

“For which point in time is the statement true that {*entity*_*or*_*property*_*span*} is {*quantity*_*span*}?”

Besides the temporal scope, we ask for the spatial scope, determination method, references, and other qualifiers in the same manner (see Table S16 for all question templates).

As the context given to the LLM may contain multiple quantities with the same surface form (that is, the same sequence of characters), the quantity that is referred to is highlighted by enclosing it in special symbols. Optionally, the property and the entity are also highlighted in the context. This approach is similar to that of the winning system in the MeasEval challenge.^15^^,^^36^

However, in contrast to the task definition of MeasEval, we also consider implicitly given properties to respect that they, in fact, are often only implicitly given. For example, the properties operating pressure and temperature are not mentioned explicitly in the statement “operating conditions are 1–20 bar and 600–1,000°C.” Similarly, humans can easily infer the quantified properties in phrases such as “20 g of platinum,” “samples were calcined for 5 h,” “poly-D-lysine (0.1 mg/mL),” or “300 mL of methanol,” even though they are not explicitly mentioned. Furthermore, in many cases, the property is indicated by an adjective (e.g., “thick” in “the 4.5-mm-thick layer” or “old” in “the 1,600-year-old tree”), but it is preferable to express the property as a noun (e.g., “thickness” or “age”). In the MuLMs corpus,^37^ 12.5% of all properties are implicit, and in our data, 47.9% of properties do not have a direct match in the text. The latter figure includes implicit properties, as well as properties for which the spelling has been corrected or the full name has been used instead of a symbol or abbreviation (e.g., “Faradaic efficiency (FE)” instead of “FE”). To account for those, we frame property extraction as generative rather than extractive QA; that is, rather than locating the answer in the given text and responding by referring to a text span, the answer is generated as free text. Therefore, the training data include an optional free-text field for specifying the implicit property, in addition to the labeled text.

Despite using a single generative model for property, entity, and qualifier extraction, only property extraction is framed as generative QA; entity and qualifier extraction are still framed as extractive QA to simplify the creation of training data and model evaluation. That is, the model generates free-text answers but is trained to reproduce text excerpts, except when the measured property is not explicitly stated. However, using a generative model enables the direct correction of typographical and parsing errors, as well as the identification of multi-part and alternative entities and qualifiers—if multiple entity spans or qualifier spans of the same type are annotated in a given example, the answer to the respective questions is the comma-separated concatenation of the annotations. Furthermore, as described in the discussion section, using generative rather than extractive QA for measured properties simplifies the use of question templates, is more likely to produce an answer at all, and acts as a “catch basin” for other details that cannot be captured by the directly extracted text spans for other concepts.

#### Quantitative statement classification

The type, rational, and system of a quantitative statement are classified using an encoder-only transformer model in a multi-label text classification setting. Type refers to the kind of claim made, rational refers to how the value was obtained, and system refers to the kind of system the claim is about. The classes were selected based on text passages describing techno-economic data for hydrogen transportation infrastructure. The classes for type are “assumption,” “feasibility estimation,” “goal,” “observation,” “prediction,” “requirement,” and “specification.” The classes for rational are “arbitrary,” “company reported,” “experiments,” “expert elicitation,” “individual literature sources,” “literature review,” “regression,” “simulation or calculation,” and “rough estimate or analogy.” The classes for system are “real world,” “lab or prototype or pilot system,” and “model.” As we have few data for this task and did not optimize the approach, we consider it only a baseline.

### Addressing the data bottleneck

As fine-tuning-based approaches for measurement context extraction have been limited by the amount of available training data,^6^ we focused on creating a large data foundation (see Table S3). Being constrained to very limited resources with regard to manually labeling large amounts of data, we used distant supervision based on Wikidata^38^ and Wikipedia^39^ to create a large dataset for measurement context extraction, called Wiki-Measurements.^40^ Additionally, we re-labeled and combined existing datasets for measurement context extraction or similar tasks to match our task design (i.e., MeasEval corpus,^36^ MSP,^41^^,^^42^ orkg-R0,^43^ PolyIE,^31^ PHEE,^44^ MuLMS,^37^ and SuperMat^45^). Finally, we manually annotated documents mainly related to hydrogen transport and power-to-X (Quinex-Hydrogen-TechData) by domain experts as part of a literature review.^46^ To re-label the existing datasets, we first heuristically applied common changes using rules and patterns.

Although quantity span identification is a less data-constrained task, we also drastically increased the amount of training data available for this task to make the models more robust against domain changes and to reduce errors at the beginning of the pipeline, which have the greatest impact due to the cascading nature of the framework. Again, we combined and re-labeled different existing datasets, manually labeled some data, and programmatically created a large dataset (see Table S1). The programmatically generated dataset is called Wiki-Quantities^40^ and is orders of magnitude larger than existing datasets for quantity span identification. It was created automatically from Wikipedia, harnessing template calls in the MediaWiki markup, similar to a previous approach.^47^ The method for creating Wiki-Quantities yielded a large amount of annotated quantities in text. However, in contrast to other quantity identification datasets, only the value and unit could be considered but no quantity modifiers. As Wiki-Quantities accounts for the majority of the data used, we adjusted the other datasets to not include modifiers rather than vice versa. Additionally, the datasets were aligned with respect to other differences in their annotation schema. The advantages and disadvantages of keeping quantity-modifier extraction separate from quantity span identification are discussed in the limitations section. The re-annotated quantity span identification datasets are the MeasEval corpus,^36^ the Grobid-quantities corpus,^11^ and the SOFC-Exp corpus.^23^

Since all datasets included errors regardless of the creation method, we manually curated a portion of the data, which we refer to as the “gold data.” The remaining data are noisier; that is, there is a higher probability that an individual record is incorrect. Hence, we refer to them as the “silver data.” Due to the effort involved in curating the data, the silver dataset is much larger than the gold dataset. To simplify data curation, we visualized the training examples using colorful emojis (see Figure S5). Their color made errors visible, and their one-character length and compatibility with basic text editors made adjustments straightforward. Common errors were quickly corrected using the editor’s search and edit features. Finally, the annotation format allows for easy online collaboration on GitHub without the need for special annotation software. As Wiki-Measurements and Wiki-Quantities are significantly larger than the other datasets, we generated additional, smaller variants by filtering out similar examples.^40^ A single person re-labeled, created, and curated the datasets. While this may aid consistency, it prevents the calculation of inter-annotator agreements.

With a total of 564,928 and 48,613 examples for quantity span identification and measurement context extraction, we drastically increased the amount of training data. In addition, the datasets cover several scientific domains and many Wikipedia topics, making the trained models applicable to a wide range of domains. The statistics on the most frequent entities, properties, and units give an indication of the primary domain of each dataset (see Tables S2 and S4). We also created a new dataset for quantitative statement classification, which, however, is small and specific to hydrogen-related energy research.

### Experimental setup and implementation

**Figure 2:**
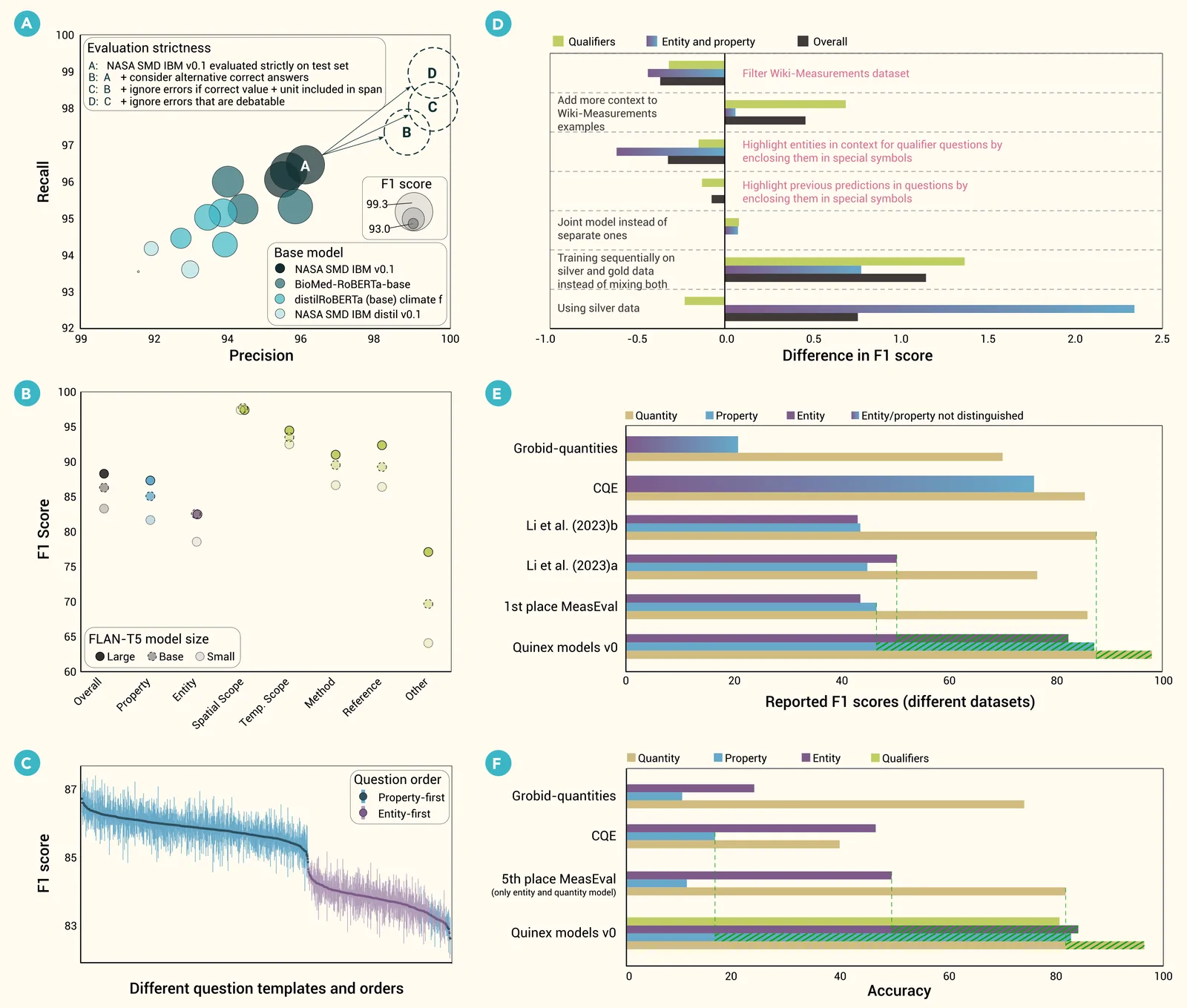
Performance of the quantity span identification and measurement context extraction models (A) Final results on the test set for different encoder-only models^56^^,^^57^^,^^58^ trained on the quantity identification task with a 50-trial hyperparameter optimization and two random seeds. Different circles for the same base model compare different training strategies (training on all data vs. silver Wiki-Quantities and then all other data) and levels of filtering Wiki-Quantities (see Table S6). To consider alternative correct answers, the best model A is manually evaluated with different levels of strictness B–D (see Table S7). (B) F1 scores on the test set for FLAN-T5 models^59^ fine-tuned on measurement context extraction. The overall values are the micro-averages. The models are first trained on all silver data and then trained on all gold data for a maximum of 20 and 35 epochs, respectively. The number of hyperparameter optimization trials per model is set relative to their training times in previous runs (see Table S12). (C) Average F1 scores on the development set for FLAN-T5-base^59^ fine-tuned on property and entity extraction for different question templates and orders. The results are averaged over three seeds and the error bars show the corresponding range of results. For more details, see Figure S3. (D) Differences in F1 score for various changes in training measurement context extraction models using a 10-trial hyperparameter optimization with three seeds (see Table S13). (E) As-reported F1 scores of different systems^11^,^15^,^36^,^42^,^60^ for quantitative information extraction (see Table S5). Different task definitions, datasets, and metrics were used. Therefore, the scores are only a rough indication of the systems’ strengths and weaknesses. (F) Entity-level accuracy of different systems^11^,^12^,^60^ for quantitative information extraction, manually evaluated on three different scientific documents (see Table 2).

All pre-trained model weights were downloaded from Hugging Face.^48^ The code for training, evaluation, and inference of LLMs was implemented using the transformers library^48^ with PyTorch.^49^ The experiments in this study involve training many models in many configurations, some of which depend on each other and are trained consecutively. To cope with this complexity and facilitate the reuse of code and the reproduction of experiments, they are defined as computational workflows using Snakemake^50^ as the workflow management system. Quantity span identification is evaluated using seqeval^51^ in the default mode. Measurement context extraction is evaluated using the SQuAD 2.0^52^ scoring script. For both, the evaluate^53^ library is used. We perform hyperparameter optimization using Optuna^54^ to maximize the F1 score on the development set. The default search algorithm is used (tree-structured Parzen estimator algorithm^55^). The algorithms for text chunking and throughput optimization for the end-to-end and application experiments are described in supplemental material and methods S1.3 and S1.4, respectively. To identify advantageous approaches to train models for quantity span identification and measurement context extraction, we compare different pre-trained language models, training strategies, and dataset compositions. The individual datasets from different sources are merged into single datasets, which are then shuffled and split into training, development, and test sets. The gold data for quantity span identification are split into training, development, and test sets in a ratio of 0.4:0.3:0.3. For measurement context extraction, the ratio is 0.7:0.15:0.15. The silver data are only used for training. All evaluations that guide decisions on how to train the final models are performed only on the development sets. The test sets are only used to evaluate the final models (see Figure 2 and Tables S6 and S12) and are not used until decisions have been made on the above aspects. We calculate the F1 score after each epoch and keep the checkpoint with the highest F1 score in the end. To prevent examples from being truncated, it is ensured that no example is longer than 512 tokens using the RoBERTa^61^ and T5^62^ tokenizers. As the random seed strongly influences the results, we repeat the experiment with different random seeds and/or perform hyperparameter optimization. The number of hyperparameter search trials and the number of runs with different random seeds averaged are reported for each experiment individually. For quantity span identification, we optimize the learning rate (between 2e−6 and 2e−4) and the number of warmup steps (0, 100, 1,000, or 10,000). The batch size is fixed at 16 unless otherwise specified. Based on preliminary experiments, we set weight decay to zero and use the AdamW optimizer^63^ with a linear learning-rate scheduler. For measurement context extraction, only the learning rate is optimized (between 1e−6 and 1e−3). The batch size for base and small models is fixed at 16 unless otherwise specified. For large models, we use a batch size of 8 with 2 gradient accumulation steps. Based on preliminary experiments, we use the Adafactor optimizer.^64^ The length of generated answers is limited to 120 tokens. If mistakes were found in the data, they were corrected. Therefore, the data are not identical between earlier and later experiments. However, the results within experiments are consistent.

## Results

The performance of current machine learning systems for extracting quantities, and especially their context, is held back by the limited amount of training data for these tasks.^6^ By addressing the data bottleneck and adjusting the task design, we achieve state-of-the-art performance on open-scope quantity span identification and measurement context extraction. Combining different approaches to dataset creation, we drastically increased the amount of data to 564,928 and 48,613 examples for quantity span identification and measurement context extraction, respectively (see the materials and methods section). In this section, the following main results are presented in detail.

• For accurate quantity span identification, small models are sufficient.
• Framing the extraction of measured properties as a generative task and sub-categorizing qualifiers seem to be important adjustments to the task design compared to the MeasEval challenge^36^ at SemEval 2021.
• In a multi-turn QA setting, extracting properties before entities is beneficial, as is incorporating previous answers into the next question templates and highlighting previous quantity predictions in the context.
• Joint models for extracting entities, properties, and qualifiers perform on par with separate models for qualifier extraction and entity-property extraction.
• Silver data improve performance only slightly despite their volume.
• Quinex performs similarly across the domains tested.
• Results from averaged, aggregated data compare well with references, but grouping entities is challenging due to different contexts or scopes.

The results are shown in Figure 2 and summarized in the following subsections. The section concludes with multiple application examples that demonstrate the practical applicability and limitations. Supplemental material and methods S3 provides additional details.

### Quantity span identification

Quantity span identification is framed as IOB sequence labeling (see the materials and methods section). Figure 2A shows the evaluation results for different encoder-only models trained on the quantity span identification task using different dataset compositions. Tables S6 and S7 provide the underlying data. The best model checkpoints per size are published as quinex-quantity-v0-124M, quinex-quantity-v0-82M, and quinex-quantity-v0-30M, respectively. The F1 score, precision, and recall of the sequence-labeling models show that small models are sufficient for accurate quantity span identification. A 124-million-parameter model achieves an F1 score of 96.27% on the test set. For context, humans previously achieved an F1 score of 95.5% in identifying quantities on the SOFC-Exp corpus, while the highest-scoring model achieved 91.6%.^23^ Other work typically achieves F1 scores in the mid to high 80s (see Figure 2E). However, as most studies include quantity modifiers within the quantity span, whereas ours does not, the results are not directly comparable.

The F1 score decreases consistently with the model’s number of parameters. Nevertheless, even the smallest model, with only 30 million parameters, still achieves 93.29% F1 on the test set. The best-performing model is based on NASA SMD IBM v.0.1^56^ (also known as Indus) and consistently outperforms the models based on BioMed-RoBERTa-base^57^ of the same size. NASA SMD IBM v.0.1 is based on the RoBERTa^61^ architecture and has been pre-trained on scientific literature using a tokenizer trained from scratch. BioMed-RoBERTa is a model derived from continuing the pre-training of RoBERTa on scientific literature. It is unclear whether the differences in performance are due to the different approaches, training corpora, or tokenizers. Table S8 contains data from preliminary experiments on a larger set of base models.

Manual evaluation of the best model’s errors shows that in many cases both the model’s answer and the ground truth are correct, although they differ (e.g., the quantities in “1.72 Ω cm^2^ and 0.31 Ω cm^2^” can be annotated as a list or individually). In other cases, it is debatable whether it should count as an error or not (is “many other parts of the world,” “bi-layer,” “quartet,” or “3D” a quantity?), and in some cases, the model is correct and the ground truth is not. Taking into account alternatively correct answers, the F1 score is 98.08%. Ignoring errors that are debatable or involve answers that are slightly too short or long but include the numeric value and unit, the F1 score is 99.25% (see Table S7).

The quality of the silver data for quantity span identification is sufficiently high that it can be mixed with the gold data without degrading performance: the differences between training on the silver Wiki-Quantities data first and then on all gold data instead of mixing both do not follow a clear pattern and are small (see Tables S6 and S9). Further experiments show that initial fine-tuning on quantity annotations from a general NER dataset neither clearly improves nor deteriorates the results (see Table S10). Likewise, randomly masking tokens in lower-quality silver data or in all data does not clearly improve results and provides inconclusive evidence of deterioration (see Table S11 and Figure S12). Although these findings demonstrate the robustness of large transformer models, the choice of random seed had a strong influence on performance, requiring the averaging of many runs with different random seeds.

### Quantity span normalization

The rule-based quantity parser is evaluated on annotated data from Grobid-quantities.^11^ The parser is evaluated on how accurately it segments quantity strings in values, units, modifiers, and uncertainty expressions; determines their type (e.g., interval); and links the units to the QUDT ontology. As the parser requires quantity modifiers such as “between” to be part of the quantity span to distinguish certain expressions of intervals from lists (e.g., “between 2 and 5” vs. “2 and 5”), the quantity spans in the Grobid-quantities corpus are expanded to include adjacent quantity modifiers. We first adapt the parser using a development set and then evaluate it on an unseen test set of different documents, yielding 91.93% accuracy (see Table S14). Adapting the parser according to the observed errors yields 96.03% accuracy, which can be considered an upper bound of the performance when tailoring the system to specific needs. The remaining errors are mainly due to the parser’s inability to correct optical character recognition and PDF parsing errors (e.g., “1mjs” instead of “1 m/s”), to distinguish units from text that should be ignored (e.g., Italy in “2% (Italy) and 4%”), to deal with unit modifiers (e.g., H2 in “kgH2” or consecutive in “consecutive days”), to perform unit disambiguation based on context rather than hard-coded priorities, and to deal with unexpected quantity expressions (e.g., “0.2 ≤ x ≤ 0.5”) or complex lists (e.g., “(ppm):7.3(1H, d, J = 5Hz), 7.2(1H, d, J = 6Hz), …”). The error categories and their frequencies are listed in detail in Table S15. The parser was reliable at self-determining whether it had failed (98.13% F1), which could be used to escalate the task to a human or LLM if the rule-based approach fails.

### Measurement context extraction

Measurement context extraction is framed as multi-turn QA (see the materials and methods section). The question prefixes are defined as templates with slots for previous predictions. Figure 2B summarizes the results of fine-tuning FLAN-T5^59^—Google’s instruction fine-tuned version of the T5 text-to-text transformer model^62^—on the measurement context extraction task, reporting overlap F1 scores as described in SQuAD.^65^ Table S12 provides the underlying data. The micro-averaged F1 scores over all questions are 88.29%, 86.31%, and 83.32% for the 783- (large), 248- (base), and 77- (small) million-parameter model, respectively. All models have higher F1 scores for identifying properties than for identifying entities (e.g., 87.32 vs. 82.49 F1 for the large model). The macro-averaged F1 over all qualifiers is 90.47% for the large model. The models are published as quinex-context-v0-783M, quinex-context-v0-248M, and quinex-context-v0-77M, respectively.

We manually analyze all predictions on the test set for measurement context extraction that do not exactly match the ground truth (351/1,853 predictions). Of these, 167 are alternative correct answers (e.g., “Curie temperature T C” vs. “Curie temperature (T C)”), some of which are preferable to the ground truth; for 58, it is debatable whether they are errors or not (e.g., “PEM electrolyzers” vs. just “PEM”), 8 are missing a special character, and 118 are clearly wrong. Considering the alternative correct answers yields an overall accuracy of 90.07%. Considering answers whose correctness is debatable or only missing a special character to be correct results in an accuracy of 93.63%.

For our dataset, the results clearly show that properties should be extracted before entities. To find advantageous question templates and determine whether to ask for the property first and then for the entity, or vice versa, we train the T5-base model on permutations of entity and property questions for both question orders. See the supplemental material and methods S5 for all tested question templates. In total, 1,080 variants are tested. The results, averaged over three different seeds, clearly show that asking for the property first yields better results than vice versa (see Figure 2B or S3A for more details). The best result was achieved by asking, “Which property or quality is characterized by {*quantity*_*span*}?” and “Which entity’s {*property*_*span*} {*is*_*or*_*are*} characterized by {*quantity*_*span*}?”

The experiments further demonstrate that incorporating previous answers into the templates for the next question is important in multi-turn QA. The property-first variants that are worse than the best entity-first variant all use the entity question “Entity:” (that is, without a slot for the quantity and property) and are all at the lower end of performance. Similarly, the property-first variants that ask for the property with “Property:” (i.e., without a slot for the quantity) are at the bottom of the performance of the property-first variants but are, interestingly, better than all entity-first variants.

Furthermore, the results show that the quantity span should be highlighted in the context (see Figure S3B). In the previous experiment (see Figure S3A), only the quantity span was highlighted by enclosing it within two asterisks to the left and right. In this experiment, we also enclose the property span in special symbols or tags when asking for the entity, hypothesizing that highlighting key information will simplify the model’s task. To find advantageous combinations, we enclose the quantity and property with a permutation of symbols and tags (e.g., ∗∗ … ∗∗, $ … $, -> … <-, **…**, <span class = “quantity”> … </span>, etc., or no enclosure at all). In total, 1,226 combinations are tested (see the supplemental material and methods S6). The results suggest that enclosing previous predictions in special symbols improves performance, with results being more conclusive for quantities than for properties (see Figure S3B). However, optimizing question templates and their order has a greater effect than optimizing enclosures. Also, in contrast to the results for quantities and properties, highlighting entities with special symbols in the context of qualifier questions showed a slightly negative effect (see Figure 2D).

Beyond that, Figure 2D shows that, although previous predictions should be incorporated into the next questions and the quantity and, optionally, the property should be highlighted in the context, highlighting previous predictions with special symbols in the questions is not beneficial. Furthermore, a joint model for extracting entities, properties, and qualifiers performs equally well as separate models for qualifier extraction and entity-property extraction. Moreover, using the silver data alongside the gold data improves performance by 2–3 F1 points for entity and property extraction. Contrary to the experiments on quantity span identification, where the silver data were of high enough quality to be mixed with the gold data (see Table S6), for measurement context extraction, this is detrimental to performance, suggesting greater differences between the silver and gold data. In particular, only the gold data include many qualifier annotations. Hence, the results of training only on gold or silver data show only a 3 F1 point difference for entity and property extraction but an 18 F1 point difference for qualifier extraction (see Table S13). Training on all the data and then on the gold data and training on the silver data and then on the gold data yield similar results.

#### Quinex outperforms related systems

By considering implicit properties and distinguishing between different qualifiers, Quinex provides a qualitative enhancement over similar systems. As our annotation schema differs from those used in other datasets (e.g., implicit properties are allowed, modifiers are not included in the quantity span, and qualifiers are sub-categorized), we cannot perform a direct, comparative evaluation. However, comparing reported F1 scores suggests a clear quantitative improvement over prior systems (see Figure 2E and Table S5 for more details). Compared to the winning system of the MeasEval challenge, Quinex yields 12, 41, and 38 higher absolute F1 points in quantity, property, and entity extraction, respectively. Nevertheless, because these systems operate under different task definitions and are evaluated using different datasets and F1 measures, the scores are only a rough indication of the systems’ strengths and weaknesses. Therefore, in the next section, we manually compare Quinex with other systems on three scientific documents, arriving at the same conclusion: Quinex yields significant accuracy improvements over similar systems. The results are visualized in Figure 2F and detailed in the next section. Note that we only compare systems that are flexible in what properties and entities they extract. Systems tailored to extract specific quantities (e.g., efficiencies of solar cells) may perform better.

### End-to-end evaluation

To assess quantity span identification and measurement context extraction end-to-end, we apply Quinex to scientific full texts from different domains and manually evaluate the results. The scientific texts comprise an energy research article on the role of hydrogen in energy systems,^66^ a medicine article on ribonucleic acid sequencing of brain tissue with regard to Alzheimer’s disease,^67^ a physics article on inertial fusion experiments,^68^ and a comment on wearable devices for health equity.^69^ We chose the articles based on their license, topic, and citation count. We did not exclude articles based on the models’ results. The scientific texts on hydrogen, Alzheimer’s disease, inertial fusion, and health devices contain 154, 497, 170, and 18 quantities, respectively. We use Grobid^70^^,^^71^ for PDF parsing. For quantity span identification, we split the full text into roughly evenly sized chunks that fit into the model’s context window. For measurement context extraction, we create the largest semantically meaningful chunk that includes the respective quantity span and fits the model’s context window when adding the question prefix (see the supplemental material and methods S1.3). Note that PDF parsing, quantity normalization, modifier identification, and quantitative statement classification are not part of this evaluation. Quinex’s predictions are evaluated manually using a specially developed web interface (see Figure S4).

The results are summarized in Table 1 and report entity-level accuracies. The micro-averaged accuracy across all classes and papers is 78.17%. Quantities, properties, and entities are identified with 92.00%, 79.31%, and 81.81% accuracy, respectively. Together, entities, properties, and quantities are correctly extracted from 69.56% of quantitative statements, but all qualifiers are correctly extracted from only 34.13% of statements. Among quantitative statements for which quantities are correctly identified, properties and entities are identified with 84.27% and 86.83% accuracy, respectively; both are correctly identified 74.81% of the time. These results demonstrate the critical dependence on correctly identifying quantities.

**Table 1:** End-to-end evaluation results of Quinex on three scientific articles and one scientific comment

	Hydrogen	Alzheimer’s	Fusion	Health devices	All papers (micro avg.)
Entity (E)	85.71 (78.6–93.8)	81.09 (70.2–88.3)	81.40 (76.7–87.4)	72.22 (61.1–86.7)	81.81 (72.9–89.1)
Entity (if quantity correct)	91.97 (83.8–94.3)	86.30 (75.2–90.6)	84.05 (78.9–87.8)	86.67 (78.6–86.7)	86.83 (77.6–90.6)
Property (P)	77.92 (73.4–87.5)	76.86 (72.4–81.4)	87.79 (84.9–94.0)	77.78 (66.7–93.3)	79.31 (75.0–85.3)
Property (if quantity correct)	83.94 (78.7–87.9)	81.58 (77.4–83.3)	91.41 (88.2–95.1)	93.33 (85.7–93.3)	84.27 (80.0–86.8)
Quantity (Q)	88.39 (87.7–97.2)	92.66 (91.3–96.1)	94.22 (93.1–97.6)	83.33 (77.8–100)	92.00 (90.7–96.7)
Quantity w/o imprecise values^a^	92.41 (91.7–98.6)	94.26 (92.8–97.3)	95.18 (94.0–98.8)	86.67 (86.7–100)	93.98 (92.8–97.8)
Quantity w/o noun/adj. units^b^	95.00 (95.0–98.7)	97.16 (96.1–97.7)	96.27 (95.5–98.5)	100.00 (100–100)	96.71 (95.9–98.0)
Quantity w/o single^c^	91.10 (90.4–97.8)	95.88 (94.4–97.1)	95.29 (94.1–98.2)	93.75 (87.5–100)	94.87 (93.5–97.5)
Temporal scope (T)	49.35 (44.2–59.0)	92.35 (92.4–96.7)	54.65 (48.3–56.9)	83.33 (83.3–100)	76.58 (74.3–81.7)
Spatial scope (S)	47.40 (37.7–56.2)	92.96 (92.8–96.5)	55.81 (45.3–57.5)	55.56 (55.6–86.7)	76.22 (72.2–81.0)
Reference (R)	85.71 (83.1–93.1)	77.46 (77.5–90.2)	83.72 (76.7–88.6)	77.78 (77.8–93.3)	80.26 (78.4–90.4)
Method (M)	64.94 (62.3–70.1)	87.53 (86.3–92.7)	87.21 (83.7–89.8)	83.33 (83.3–100)	83.23 (81.3–88.2)
Other qualifiers (O)	55.19 (43.5–63.9)	56.14 (39.2–73.3)	56.98 (53.5–59.3)	38.89 (27.8–46.7)	55.77 (42.7–68.2)
Overall (micro average)^d^	69.34 (63.8–77.6)	82.15 (77.8–89.4)	75.24 (70.3–78.9)	71.53 (66.7–88.3)	78.17 (73.5–85.1)
<E, P, Q> together	69.48 (59.1–81.9)	67.81 (55.1–75.4)	74.42 (66.3–83.2)	72.22 (50.0–86.7)	69.56 (58.0–78.4)
<E, P, Q> together (if Q correct)	78.10 (66.9–83.7)	72.16 (59.6–77.3)	78.53 (70.8–84.8)	86.67 (64.3–86.7)	74.81 (63.3–80.2)
<T, S, R, M, O> together	22.08 (14.3–30.6)	43.06 (30.8–62.4)	20.93 (16.3–22.8)	16.67 (11.1–33.3)	34.13 (24.4–48.0)
<T, S, R, M, O> together (if Q correct)	24.09 (16.2–30.5)	45.82 (32.4–64.0)	21.47 (16.1–23.2)	20.00 (14.3–33.3)	36.45 (25.8–48.9)

For each class, we report the entity-level accuracy, that is, the ratio between the correctly predicted ones and the correctly plus incorrectly predicted ones. If a quantity is not recognized, all classes are considered incorrect for that quantity. If a non-quantity (e.g., a postal code or ID) is identified as a quantity, that quantity is considered incorrect, but the other classes are not evaluated. The first score represents the best judgment. The numbers in parentheses represent the scores for strict and relaxed evaluation. These are calculated by setting all judgments of low confidence to “incorrect” and “correct,” respectively.

a Several regions, multi-view, etc.

b 5 regions, two-sided, etc.

c Single void, single gene.

d Considering rows E, P, Q, T, S, R, M, and O.

Of the 839 quantities in the documents, 41 were false negatives, 11 were inaccurately labeled, and an additional 8 were false positives. Most of the 60 incorrect quantity predictions concern quantities with “single” as the value, such as “single-gene” (22%). The next most common incorrect predictions concern quantities with “many,” “multiple,” or “multi-” as the value (17%), followed by non-quantities mistaken as quantities (e.g., product IDs, years, or line numbers; 10%). Other incorrect predictions concern quantities with “one” as the value, where it can be an article (e.g., “one method”; 7%); quantities with “double,” “doubles,” or “doubled” as the value (5%); relative quantities, for which the reference quantity should be included in the quantity span but is not (e.g., “six times that of today’s total number of ships on this route”; 5%); and quantities for which the predicted span is slightly too short or long (5%). Excluding errors involving imprecise quantities, quantities with the value “single,” and quantities with noun or adjectival units, the accuracy in identifying quantities is 93.98%, 94.87%, and 96.71%, respectively.

The document on health devices contains only 18 quantities, of which “single patient,” “single, wireless probe,” and “many devices” were not identified, resulting in a comparably low accuracy. Property extraction was more accurate for the fusion article than for the others. Otherwise, the quantity, property, and entity extraction results are fairly consistent across domains. Qualifier scores vary more widely for two reasons: first, depending on the text, certain qualifiers are more or less frequent (e.g., quantitative statements in the article on Alzheimer’s disease did not have a spatiotemporal scope, making their extraction easy). Second, information provided in one paragraph remained relevant throughout the article but was lost due to the limited 512-token window. For example, in the fusion article, the spatial scope (the location of the facility) and the temporal scope (based on an explanation on how to decode the date of a shot based on its identifier) are only given once; in the Alzheimer’s disease article, on two occasions, a reference is given that is valid for a longer section of text; and in the hydrogen article, the spatiotemporal scope and method were given at the beginning of the article but were mostly not part of the context for later sections. In the article on Alzheimer’s disease, the manufacturer and catalog number of the products were often mistakenly extracted as references (e.g., “Thermo Fisher, N7805100” in “primary human astrocytes (Thermo Fisher, N7805100)”). Furthermore, many quantitative statements require contextualization within a longer experimental procedure to be meaningful (e.g., the concentration of a certain substance in a solution), which the qualifiers in Quinex provide only to a limited extent.

We ran Quinex again on the hydrogen-related article, this time prefixing the local context with a sentence summarizing the article, to test whether this would allow the model to keep track of the spatiotemporal scope and method throughout the article. Indeed, the accuracy of the spatiotemporal scope and method is significantly higher, albeit at the cost of reduced accuracy in extracting entities and properties. Comparing predictions with and without distant context shows that there are often multiple correct solutions in the open information extraction approach to measurement context extraction. For example, the <entity, property, quantity> triples <LH2 transportation, share of LH2 transported by trucks, 4 TWh/a> and <Trucks, contribution to the LH2 transportation, 4 TWh/a> are both correct. Similarly, <hydrogen pipelines, increase in total length, 7%> and <national greenhouse gas-neutral energy supply system for Germany, increase in total length of hydrogen pipelines, 7%> are both correct.

Using the same documents, Table 2 and Figure 2F compare the performance of Quinex with Grobid-quantities,^11^ Counts@IITK^12^ (a MeasEval entry), and CQE.^60^ These systems were selected based on the availability of their code and models. Note that only the quantity and entity models of Counts@IITK are used because the property and qualifier models are not published. Since Quinex is based on domain-specific training data for hydrogen technologies, the hydrogen article was excluded from this evaluation. Additionally, to evaluate the systems more independently of design decisions regarding what constitutes a quantity, debatable and imprecise quantities are ignored, except when calculating the lower bound of performance. Furthermore, since CQE and Grobid-quantities do not distinguish between entities and properties and since we only have access to the entity model for Counts@IITK, the baseline systems are favored in several ways when evaluating entity and property extraction: first, we mark confusions between entities and properties as low-confidence correct rather than incorrect. Therefore, they are only considered incorrect at the lower bound of the score. Similarly, for counts (e.g., “three sensors”), empty property predictions are treated as low-confidence correct rather than incorrect. Lastly, when property predictions are empty but the property can be inferred from entity and quantity predictions, the absence of a property prediction is marked as low-confidence incorrect. In other words, they are considered correct at the upper bound. This is generous because inferring the property from the entity and quantity is generally misleading. For instance, “tubes” and “400 g” in the Alzheimer’s paper suggest mass, yet they refer to the relative centrifugal force applied. Furthermore, we do not penalize incomplete entity extraction (e.g., “tagment DNA enzyme” instead of “tagment DNA enzyme (Nextera, Illumina)”), despite the lack of qualifier extraction to capture such details. Quinex’s predictions are often more meaningful and contextualized by additional qualifiers. The benchmark script, scoring script, and judgments are published in the Quinex repository.

**Table 2:** End-to-end performance comparison of Quinex and three other systems (Grobid-quantities, CQE, and the MeasEval entry Counts@IITK) on scientific documents

Paper	System	Entity (E)	Property (P)	Quantity (Q)	Overall (micro average)	<E, P, Q> together
Fusion	Quinex	84.34 (76.7–88.0)	90.96 (84.9–94.6)	97.60 (93.1–98.2)	90.98 (84.9–93.6)	77.11 (66.3–83.7)
	Counts@IITK	39.76 (30.9–41.6)	19.28 (0.0–24.7)	70.65 (58.2–71.6)	45.03 (31.5–47.7)	0.00 (0.0–4.2)
	Grobid-quantities	9.15 (5.6–13.4)	18.90 (0.0–25.6)	63.83 (50.7–63.8)	32.17 (20.2–35.7)	0.00 (0.0–1.8)
	CQE	31.55 (23.6–35.1)	29.17 (7.3–35.1)	28.32 (26.9–30.1)	29.26 (21.5–32.4)	8.33 (2.8–16.1)
Health devices	Quinex	86.67 (61.1–86.7)	93.33 (66.7–93.3)	100.00 (77.8–100)	93.33 (68.5–93.3)	86.67 (50.0–86.7)
	Counts@IITK	46.15 (33.3–46.2)	53.85 (0.0–53.8)	81.25 (42.9–81.2)	61.90 (26.3–61.9)	15.38 (0.0–15.4)
	Grobid-quantities	35.71 (21.1–35.7)	50.00 (0.0–50.0)	87.50 (47.6–87.5)	59.09 (23.7–59.1)	21.43 (0.0–21.4)
	CQE	50.00 (36.8–56.2)	56.25 (0.0–68.8)	72.73 (60.0–72.7)	61.11 (34.9–66.7)	25.00 (0.0–37.5)
Alzheimer’s	Quinex	84.13 (70.2–88.3)	79.75 (72.4–81.4)	96.09 (91.3–96.1)	86.70 (78.0–88.6)	70.35 (55.1–75.4)
	Counts@IITK	52.84 (41.1–58.5)	7.16 (0.0–30.5)	86.40 (73.0–88.2)	49.45 (38.6–59.6)	3.16 (0.0–22.1)
	Grobid-quantities	28.36 (24.2–32.3)	6.21 (0.0–29.0)	77.38 (61.9–78.7)	38.71 (29.8–47.8)	1.66 (0.0–17.0)
	CQE	51.44 (38.4–57.0)	10.70 (4.7–17.9)	45.36 (39.5–48.6)	36.83 (28.7–41.9)	4.73 (2.2–10.9)
All (micro avg.)	Quinex	84.24 (71.6–88.2)	82.88 (75.4–85.0)	96.56 (91.4–96.7)	87.93 (79.5–90.0)	72.42 (57.8–77.7)
	Counts@IITK	49.39 (38.3–54.0)	11.16 (0.0–29.5)	81.87 (68.0–83.4)	48.54 (36.4–56.5)	2.60 (0.0–17.4)
	Grobid-quantities	23.75 (19.4–27.7)	10.29 (0.0–28.6)	74.15 (58.6–75.1)	37.51 (27.2–45.0)	1.66 (0.0–13.3)
	CQE	46.42 (34.7–51.5)	16.42 (5.2–23.4)	39.68 (35.4–42.3)	35.09 (26.7–39.6)	6.12 (2.3–12.8)

For each class, we report the entity-level accuracy as described in Table 1. Note that for Counts@IITK, only the quantity and entity models are used, as the property and qualifier models are not published.

Similar to the comparison of systems using self-reported F1 scores, Quinex clearly outperforms related systems. Quinex yields 14.69%, 66.46%, and 34.85% higher accuracy in quantity, property, and entity extraction, respectively, than the next-best-performing system in each category. Additionally, Quinex performs substantially more robustly across the papers, whereas the performance of the other systems varies widely. When comparing the accuracy of correctly identifying complete sets of entities, properties, and quantities, Quinex achieves 66.3% higher accuracy than CQE despite manually classifying CQE’s answer spans as entities and properties. Grobid-quantities and Counts@IITK have lower scores because they provide only a single span for the measured concept; therefore, the entity and property can both only be correct if an empty answer is considered correct (e.g., for counts). Despite being rule based, CQE performs well against other baselines in entity and property extraction. However, it performs considerably worse in quantity span identification, frequently mistaking figures, tables, and literature references for quantities.

### Application experiments

**Figure 3:**
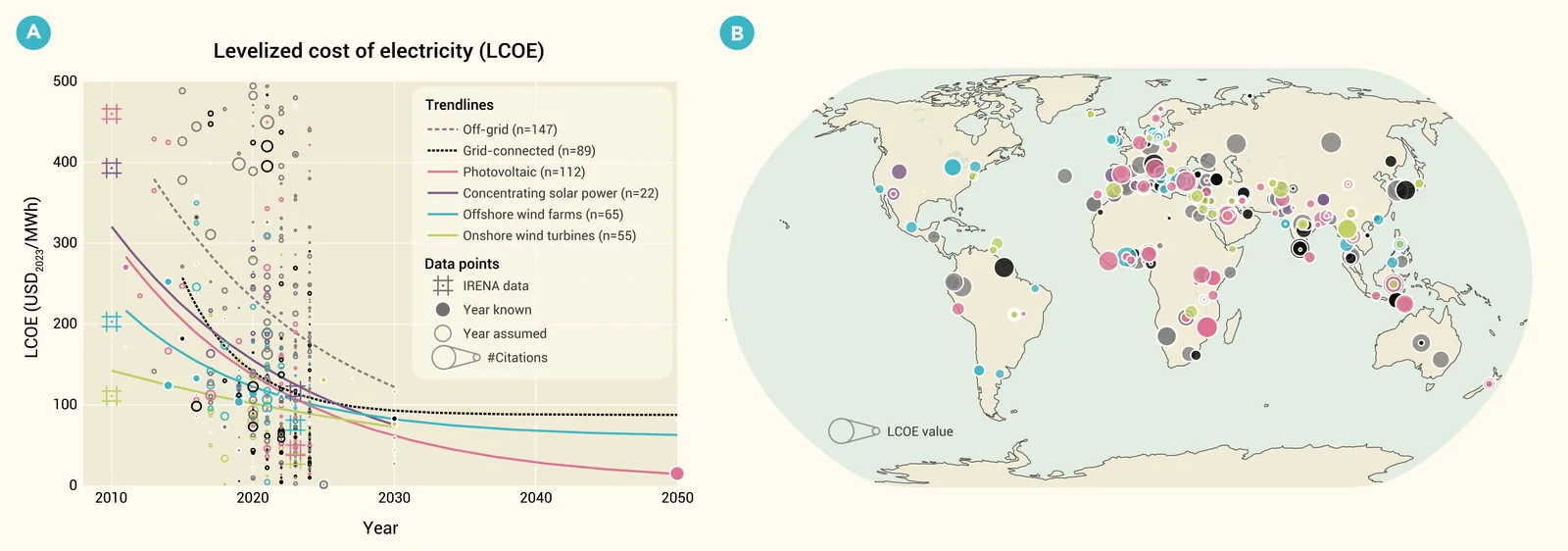
Automatically extracted LCOE values for different energy technologies The LCOE values are displayed relative to their (A) temporal and (B) spatial dimensions, with IRENA data^72^ for reference. 17,680 quantitative statements were identified in 2,383 abstracts. After filtering for LCOE values for the technologies and temporal scope visualized in (A) and ignoring unrealistic values that are negative or higher than 500 $_2023_/MWh, 490 data points from 471 unique statements remained. Further filtering for statements that could be assigned latitude and longitude resulted in 328 data points, which are visualized in (B). All units are converted to $_2023_/MWh, accounting for inflation and currency exchange. If the temporal scope is unknown, the publication year is assumed (unfilled circles). Trendlines are created by fitting a positive exponential decay to the data. Data points for which the temporal scope is known are weighted higher (5×), as are highly cited papers (1×–5×).

**Figure 4:**
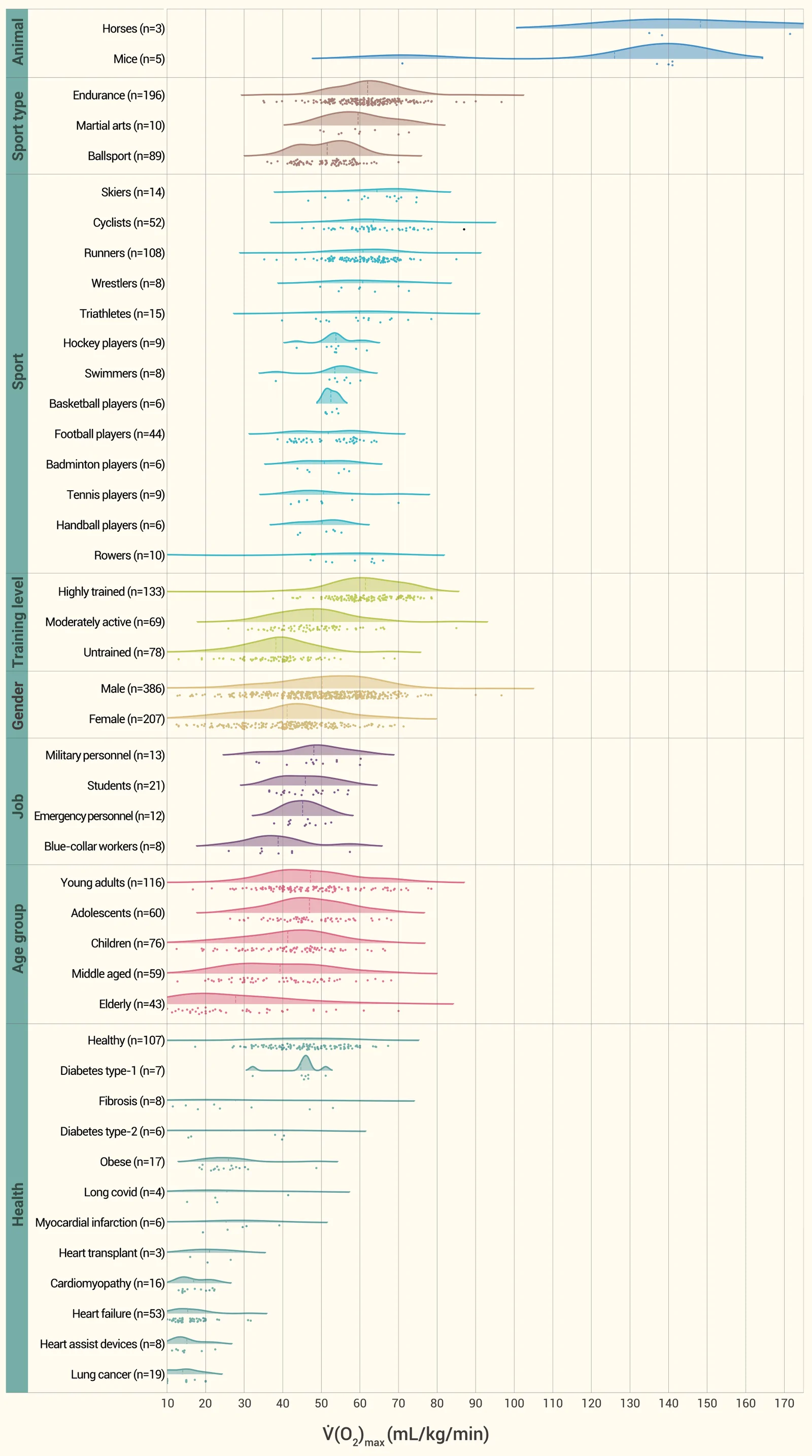
Automatically extracted maximal oxygen uptake ($\dot{V}$O_2_ max) distributions 42,794 quantitative statements were identified in 2,490 abstracts. After filtering for $\dot{V}$O_2_ max and the considered categories, ignoring unrealistic values outside [0, 500] mL/kg/min, and manually checking the few remaining values outside [5, 100] mL/kg/min, the 2,133 data points from 1,133 unique statements visualized here remained. For each category, the number of included data points (*n*) is given.

**Figure 5:**
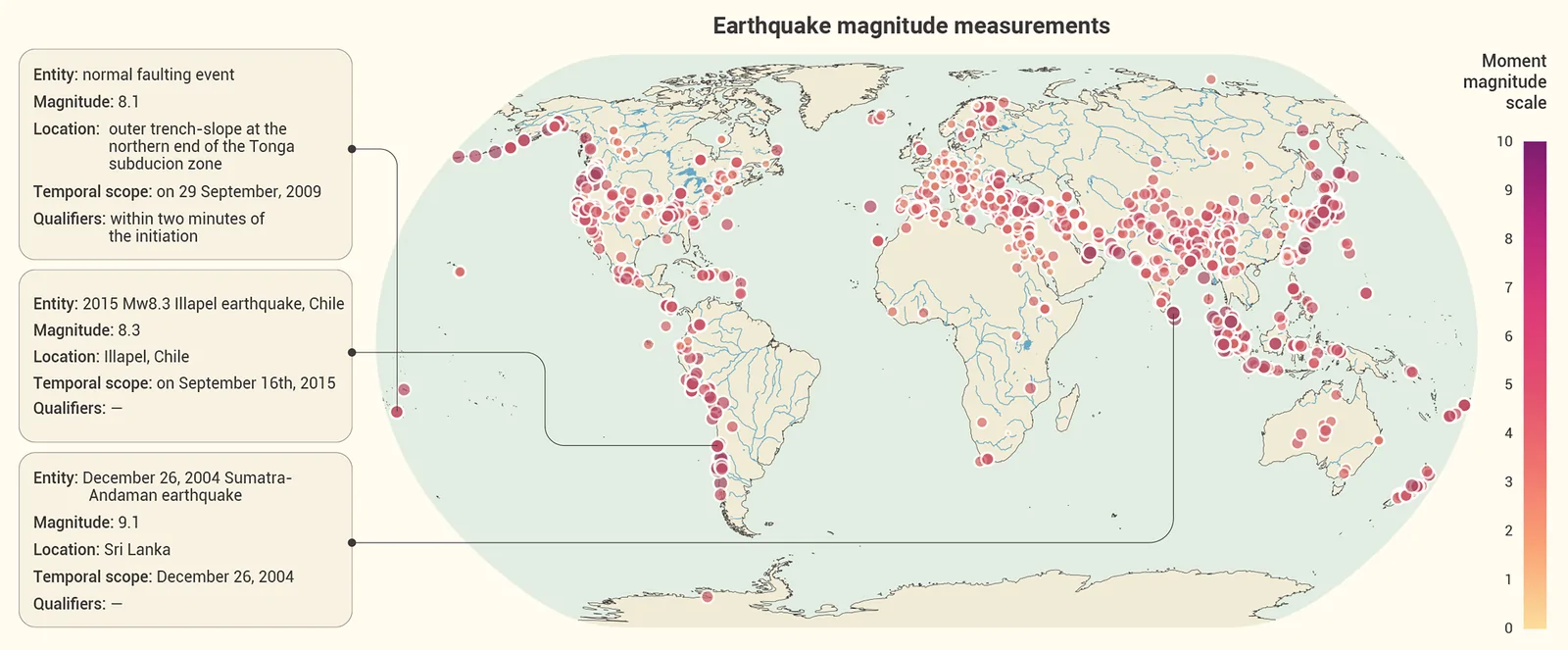
Automatically extracted moment magnitudes of earthquakes relative to their epicenter or measurement location 26,696 quantitative statements were identified in 3,631 abstracts. After filtering for moment magnitudes and valid latitudes and longitudes and ignoring unrealistic values that are negative or higher than 10.6, the 1,690 statements visualized here remained.

**Figure 6:**
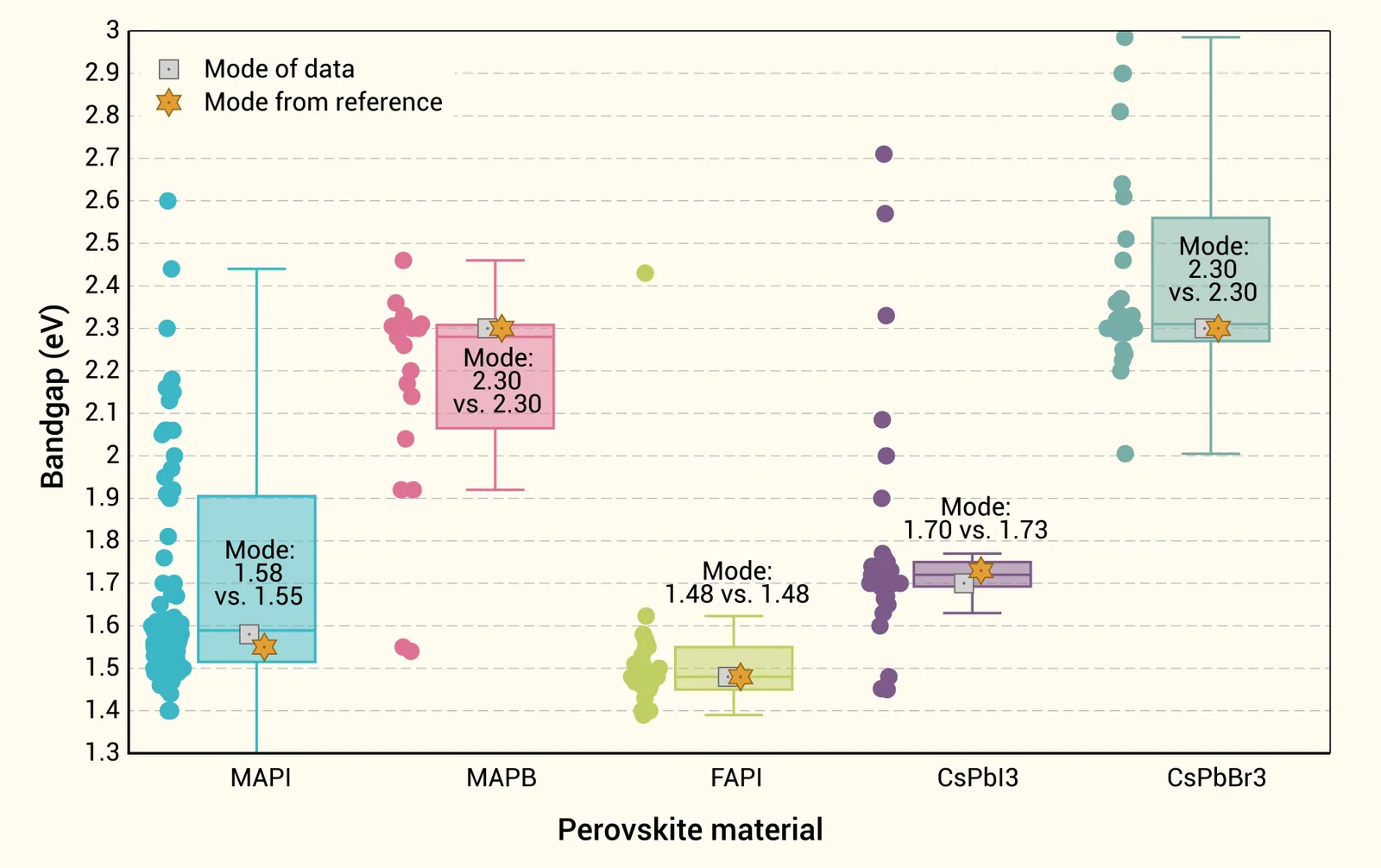
Automatically extracted band-gap distributions of different perovskite materials Mode values of the data extracted by Sipilä et al.^27^ are shown for reference. 4,710 quantitative statements were identified in 828 abstracts. After filtering for band-gap values for the considered materials and ignoring unrealistic values that are negative or higher than 5 eV, the 988 statements visualized here remained.

To test Quinex’s usefulness in practical applications, we apply it to collecting values for the levelized cost of electricity (LCOE) for different energy technologies, maximal oxygen uptakes ($\dot{V}$O_2_ max) of humans and animals, moment magnitude measurements of earthquakes, and band gaps of perovskite materials (see Figures 3, 4, 5, and 6). For this, we query Scopus for abstracts likely to mention the respective values, process the abstracts with Quinex, and filter and normalize the data for visualization. The numbers of abstracts and quantitative statements considered are given in the respective figure captions. The queries are provided in the supplemental material and methods S7. Note that Quinex does not need to be modified for each experimental setup. The pipeline was used as is; only the normalization of entities, properties, and qualifiers required manual effort, as their normalization is not yet part of Quinex. Using a single quantity model and three context models in parallel, processing the abstracts one after the other yielded an average execution time per quantitative statement between 0.4 and 0.5 s on an NVIDIA A40 GPU, without optimizing batch sizes or the number of parallel models. We filter and categorize the extracted quantitative statements based on keywords. Intervals are reduced to single values by taking the average unless the temporal scope is specified individually for the bounds. The temporal scope is normalized using rules (e.g., 2020 for “at year 2020,” the publication year for “currently,” and the publication year plus 10 years for “mid-term future”). If the temporal scope is unknown, the publication year is assumed. The spatial scope is normalized by prompting an LLM (Qwen3-30B-A3B-Instruct-2507^73^) to return geo coordinates. We sample two geo coordinate predictions and drop them if they differ by more than 300 km.

Figure 3 shows the LCOE of different energy technologies and systems relative to their temporal (Figure 3A) and spatial (Figure 3B) dimensions. The LCOE is a measure of the cost of a generator (i.e., power plant) in the electricity grid, which relates the total cost of constructing and operating the generator to the electricity produced over its lifetime. The lower a technology’s LCOE, the cheaper its electricity generation. Hence, it is a key characteristic for analyzing energy transition pathways. Data from the International Renewable Energy Agency (IRENA) is used as a reference.^72^ The price of renewable energy technologies decreased steeply and is expected to continue to do so. The orders of magnitude, rankings, and trends are well captured, considering that the system is not tailored to extract LCOE data and that the entity classes include technologies of different scales (e.g., rooftop photovoltaics vs. solar parks).

Figure 4 shows the $\dot{V}$O_2_ max distribution of subjects of different categories (animal, sport type, sport, training level, gender, job, age group, and health status). $\dot{V}$O_2_ max is a measure of the maximum volume flow of oxygen a body can utilize in physical exertion and is an indicator of endurance performance. The results make intuitive sense, and spot checks confirmed their accuracy. However, as this is not our field of expertise, please treat the results with caution.

Figure 5 shows the moment magnitude of earthquakes relative to their epicenters or measurement locations. The results resemble similar map visualizations^74^ but show more locations on the west coast of the USA and northern Europe, perhaps because scenario values are also included.

Figure 6 shows the band-gap distributions of different perovskite materials. Band gaps of perovskites are important for solar cell research. To assign the results to the different materials, we used the synonyms given in previous research.^27^ The mode values of the distributions closely match those extracted automatically previously, which were found to align well with experimental results.^27^

## Discussion

The application experiments demonstrate that today’s quantitative information extraction is of sufficient quality to be of practical use—even when using smaller-scale models—and can generalize well across domains. The comparatively low computational requirements and generalizability make Quinex applicable for quantitative information extraction on a large scale for a wide audience. Potential use cases include automated literature reviews, quantitative search, trend monitoring, and database population—tasks that are common to many research fields and beyond but typically performed in a manual, labor-intensive manner.

Quinex showed substantial performance gains compared to other systems in the literature. We assume that most of this can be attributed to a larger training corpus and the different task design. We do not consider modifiers part of the quantity spans and frame property extraction as a generative QA task. Although the latter increases the space of possible answers, in many cases, they are more intuitive. For example, instead of the <entity, property, quantity> triples <metal, pure, 99.9%> or <vessel, heated, 1,023 K> in MeasEval, a generative setting allows for the properties “purity” and “heating temperature” instead. The generative setting is also advantageous for multi-turn QA: first because properties can be easily inserted into templates to form grammatically correct sentences (e.g., “Which entity’s purity is characterized by 99.9%?” instead of “Which entity’s pure is characterized by 99.9%?”) and second because generative QA is more likely to produce an answer at all, as many properties are implicit. Furthermore, the generated text for the measured property not only accounts for implicit properties but also acts as a catch basin for other details that cannot be captured by the text spans for quantities, measured entities, and qualifiers. However, framing property extraction as a generative task leads to hallucinations. Properties that occur frequently in the training data (see Table S4) are more likely to be hallucinated. While only a few hallucinations occurred in the end-to-end experiments, in the $\dot{V}$O_2_ max application experiment, *p* values were sometimes mistakenly considered to be basic reproduction number estimates (R0). Currently, other than training the model to prioritize text excerpts as answers when available, we do not employ any methods to counter or detect hallucinations. Nevertheless, hallucinations are often easy to spot based on the entity involved.

Due to their statistical nature, we do not place too much emphasis on the exact choice of questions and enclosures; if we continue the experiments for more random seeds or choose a different base model, the results may favor another option. Nevertheless, the results are clear that the quantity should be highlighted in the context and that the property should be asked for before the entity is asked for. The quantity must be highlighted to avoid confusion with other possible identical quantities. Asking for the property first may be advantageous because it seems more natural in language usage not to skip the property that connects the entity and the quantity, and because their lower diversity in the training and test data makes them likely easier to identify than entities (see Table S4). In addition, asking for the property first makes the second question more likely to be grammatically correct, as articles do not need to be considered and, unlike properties, entities can be objects, persons, processes, and so on. Finally, it is probably clearer what is meant by property/attribute/ … than by entity/item/ …, which reduces the chance of confusion. A reason why highlighting the quantity is more important than highlighting the property is that, unlike the property, the quantity is highlighted in each question’s context, and it is important to know exactly which quantity is being referred to in order to extract the measurement context.

Subdividing qualifiers into more specific classes (e.g., temporal scope, references, and methods) allows for more targeted questions. Questions such as, “For which point in time is the statement true that … ?” are easier to answer than more open-ended questions such as, “Under which constraints is the statement true that … ?” This may be reflected in the lower scores for the generic qualifier class on both the test set and in the end-to-end evaluation, but the difference could also be because an empty answer is more often the correct answer for the specialized qualifier questions than for the generic one. The share of examples in the test set with non-empty temporal scopes, spatial scopes, references, methods, and other qualifiers is 22%, 8%, 34%, 24%, and 52%, respectively. Qualifier extraction was deemed the most difficult task in the MeasEval challenge, with the winning system achieving only 16.3% SQuAD overlap F1, and even humans struggled to agree on it during annotation (0.334 Krippendorff’s alpha).^36^ While Quinex performs surprisingly well in qualifier extraction, achieving 90.47% macro-averaged SQuAD overlap F1, the subdivision of qualifiers and averaging of their scores artificially raises Quinex’s scores. Accordingly, in the end-to-end evaluation, all qualifiers were correctly extracted for only 34.13% of quantitative statements. We observed that distant qualifiers were often not included in the 512 tokens given to the model. Therefore, future work should use LLMs with larger context windows (e.g., more recent models or long-context transformers with sparse or efficient attention) and algorithms for selecting relevant context (e.g., approaches based on retrieval and re-ranking and/or hierarchical summarization).

Currently, Quinex does not cluster entities, properties, or qualifiers, nor does it link them to ontology classes. Therefore, the extracted data must be processed further to enable aggregations and visualization, such as in the application examples. Grouping entities is particularly challenging as different contexts or scopes can make them difficult to compare.

Furthermore, the rule-based quantity parser cannot disambiguate units based on the context and fails on unexpected quantity expressions (e.g., due to PDF parsing errors or the presence of unit modifiers). For this reason, and because each new rule makes the parser more complex, switching to a machine-learning-based approach seems advantageous, at least for more complex and ambiguous cases. However, as the parser’s rules for predicting the correctness of its output were accurate, a hybrid approach may be most efficient.

As the model predictions are not error free, we suggest use cases with human oversight or that do not require highly accurate data, such as information retrieval or larger statistical analyses (see responsible use section). Collecting more curated gold data, of which we had few, and fine-tuning toward specific use cases will improve results further. To collect more data, we will use the Quinex web interface (see Figure S4) and intend to update the training data and models regularly. Furthermore, we hope for online collaboration in the creation and curation of training data. This is facilitated by the annotation format shown in Figure S5. Collecting more data from its application in different scientific domains is crucial for improving Quinex, as our results show that the effect of heuristically labeled data is limited despite their volume. In quantity span identification, the best performance was achieved by reducing the size of Wiki-Quantities. In measurement context extraction, training on the full Wiki-Measurements dataset rather than a smaller variant improved performance only modestly. This is likely because the Wikipedia-based datasets are much larger than all other datasets used for training, but the development and test data do not reflect this imbalance to the same extent. This also shows that the Wikipedia-based datasets are too simple or not diverse enough for the models to generalize well to more complex, hand-labeled data from scientific papers. If we were to start from scratch, we would focus more on manual labeling and use multi-billion-parameter LLMs to generate initial training data instead of creating them heuristically. However, for quantitative information extraction, Quinex can now be used as a baseline for pre-annotating data. In fact, we already used Quinex to spot mistakes in the datasets, even in the human-curated data. Combining datasets from different sources, we found that they all initially contained errors, regardless of how they were created.

Quinex uses a two-step approach of identifying all quantities prior to the measurement context. This approach is useful if the entities or properties to extract are not known beforehand or if their number is large. Because the models for quantity span identification can be much smaller than those required for measurement context extraction, the two-step approach is especially advantageous if there are few quantities relative to text length. However, if the entities and properties for which quantitative data are sought are known, their number is small, and there is little text to analyze, then simply prompting an LLM, “What is the numerical value of {*property*} of {*entity*}?”^27^ is likely the preferable approach. Likewise, if capturing implicit quantities is a priority, this approach is preferable because sequence labeling cannot capture them. Although rare for physical quantities, sometimes a quantity is implicit and requires world knowledge and reasoning, such as counting. For example, the number of elephants and puffins in the zoo described in the sentence “the zoo is very tiny hosting only an elephant and a puffin” is one each; in “after a failed first attempt, she succeeded in summiting the mountain on her next run,” the number of attempts is two; and in “all the wheels of the car are driven,” the number of driven wheels is likely four. The quantity span identification model, however, would fail to recognize these quantities. By reversing the approach and asking directly for the quantity of interest (e.g., “What is the number of elephants in the zoo?”), implicit quantities can be captured, yet the measured entity and property of interest must be known beforehand. Ultimately, Quinex addresses a specific information requirement. If requirements deviate from this, the fine-tuning approach makes Quinex less adaptable, in which case using the in-context learning capabilities of larger LLMs may be preferable. In some cases, a combination of approaches can be preferable. For example, using Quinex, text passages without quantities can be skipped, and documents can be efficiently indexed for quantities and, optionally, their measurement context to improve the relevance of the documents retrieved, e.g., for numerical questions in retrieval-augmented generation applications.

### Responsible use

Given the high level of interest in automated data collection across many domains, including regulatory and high-impact research areas, it is essential that researchers understand the limitations of Quinex and use it responsibly. Humans tend to be overly confident in AI.^75^^,^^76^ It is therefore important to emphasize that Quinex makes errors. Without manual validation, the automatically collected data cannot be trusted, and publishing them without clearly communicating the presence of errors would be negligent. Not only could it mislead its recipients, but it could also undermine trust in science.

Consequently, we recommend use cases in which Quinex informs its users, who remain responsible for verifying and interpreting the results. For example, providing an overview of the quantitative statements in a research area enables researchers to explore it and discover interesting data, literature, and trends. However, users are responsible for verifying the data based on their source. The approach taken in Quinex ensures that the exact source and text passage can always be traced back. To promote verification, we display the corresponding excerpts beside the data in the dashboard and allow users to jump to the exact locations in the source documents.

Statistical analyses of aggregated data can reduce the risk of drawing incorrect conclusions from a small number of individual data points. However, a balance must be struck between minimizing the impact of individual errors and maintaining the traceability of the results. Biases in the models may skew the overall results. Even when validating extracted data based on their sources, models may systematically miss certain types of data or expressions. In particular, Quinex does not yet consider tables and figures. Additionally, large-scale analyses will be inherently biased by what literature can be accessed automatically.

Lastly, we recommend testing the reliability of Quinex in your particular domain and topic of interest to avoid placing too much confidence in the aggregated results. If you notice any behavior that could distort the aggregated data or mislead researchers, please contact the authors of this article. While we have shown state-of-the-art performance with Quinex, we advise against placing too much reliance on automatic data collection methods. Responsible use acknowledges and clearly communicates their error proneness.

### Limitations

The study design and methods used have several limitations. The study was primarily centered on quantity span identification and measurement context extraction, with only a brief mention of statement-type classification and quantity normalization. The quantity parser relies on hard-coded rules, including for unit disambiguation. However, the parser is published open-source and can easily be extended. As quantitative statement classification gives poor results with the little data currently available for this task, we will collect more data in the future.

Our study does not include formal significance testing or comprehensive statistical comparisons with previous systems because our task formulation differs from earlier work. Nevertheless, we present extensive empirical evidence to support our findings.

In the end-to-end evaluation, we consider alternative correct answers, but users may prefer one over the other. Furthermore, qualifiers can compensate for details omitted in entity and property predictions, making it subjective in some cases whether the entity/property or qualifier lacks detail.

Keeping the quantity modifiers separate from the quantity spans is a reasonable approach, as the quantity span primarily serves as an anchor for measurement context extraction. Furthermore, the inclusion of quantity modifiers would make filling the question templates for measurement context extraction more prone to grammatical mistakes (“… is approx. 6 kW?” ✓ vs. “… is reach 6 kW?” ✗), and quantity modifier extraction also has to consider those that are not adjacent or inside a quantity span anyway. Nonetheless, in isolation, without performing the additional step of quantity modifier extraction, the quantity spans given by the quantity span identification model are less meaningful and less comparable to prior work.

In addition, using the T5 tokenizer without modification, the responses of the model for the extraction of the measurement context miss special symbols such as Greek letters (e.g., *λ*, *ϵ*, or Δ). Also, some of the symbols tested for enclosing previous predictions are unknown to the tokenizer. We will address this limitation in future releases. Quantity span identification is not affected by this.

Furthermore, the measured entity is sometimes best referred to by multiple, non-contiguous spans (e.g., “polymer P3” in “The polymer that performed best, P3, exhibited a …”). Although we now account for this, we did not do so initially; hence, it is not fully reflected in the training data.

Quinex is highly accurate in identifying physical quantities (96.71% for quantities without noun phrases or adjectives as units). However, it does not reliably identify imprecise quantities (e.g., “many devices”) or quantities expressed as “single …” or “double …” This is because these were not the focus of this work and are not consistently annotated in the training data.

The large effect of the random seed in fine-tuning transformer models has been noted many times in the literature (e.g., Dodge et al.^77^) and was also found in this study. For example, when optimizing the enclosures to highlight previous predictions in this context, the mean difference between the best and worst F1 scores using different seeds but the same special symbols is 0.69 F1 points, the standard deviation is 0.34, and the minimum and maximum values are 0.01 and 1.90 F1 points.

The configuration in the first row of Table S13 was considered optimal based on preliminary results on the development set and is used to train the models for the end-to-end evaluation and the application experiment. However, when the experiment was repeated with more random seeds and evaluated on the test set, the results suggest that a different configuration is slightly better, specifically: entities should not be highlighted in the context for qualifier questions, and the Wiki-Measurements dataset should not be filtered and used in a variant with more context surrounding the quantity. We will implement these changes in the next release of Quinex.

## Resource availability

### Materials availability

This study did not generate new unique materials or reagents.

### Data and code availability

The models, most datasets, and the code are published openly. The models are available at https://huggingface.co/collections/JuelichSystemsAnalysis/quinex-v0. The models for quantity span identification are published in different sizes: base (124 M), small (82 M), and tiny (30 M). Likewise, the models for measurement context extraction are published in large (783 M), base (248 M), and small (77 M) sizes. The datasets are available at https://github.com/FZJ-IEK3-VSA/quinex-datasets, the code for Quinex is available at https://github.com/FZJ-IEK3-VSA/quinex, and the code for the rule-based quantity parser is available at https://github.com/FZJ-IEK3-VSA/quinex-utils.

## Funding and acknowledgments

The authors would like to thank the German federal government, the German state governments, and the Joint Science Conference (GWK) for their funding and support as part of the NFDI4Ing consortium. This work was funded by the 10.13039/501100001659German Research Foundation (DFG) – project number: 442146713. Furthermore, this work was supported by the 10.13039/501100009318Helmholtz Association under the program “Energy System Design.” The funders had no role in the study design, data collection and analysis, decision to publish, or preparation of the manuscript.

## Author contributions

J.G. conceived the methodology and experiments, analyzed the results, and wrote the original draft. J.G., G.M., and L.L. created the datasets. J.G. and C.K. created the web service and evaluated model predictions. H.K. managed the infrastructures for high-performance computing. P.K., J.M.W., and D.S. took care of the supervision, project administration, and funding acquisition. All authors contributed to the manuscript and approved the final version.

## Declaration of interests

The authors declare no conflicts of interest.

## Supplemental information

Document S1. Figures S1–S5, Tables S1–S16, and supplemental material and methods

Document S2. Article plus supplemental information

## References

[1] Reimer L.C., Sardà Carbasse J., Koblitz J. (2022). BacDive in 2022: the knowledge base for standardized bacterial and archaeal data. Nucleic Acids Res..

[2] Jacobsson T.J., Hultqvist A., García-Fernández A. (2021). An open-access database and analysis tool for perovskite solar cells based on the FAIR data principles. Nat. Energy.

[3] Reinhard J., Wernet G., Zah R. (2019). Contribution-based prioritization of LCI database improvements: the most important unit processes in ecoinvent. Int. J. Life Cycle Assess..

[4] Shiraki H., Sugiyama M. (2020). Back to the basic: toward improvement of technoeconomic representation in integrated assessment models. Clim. Change.

[5] Hatton L., Johnson N., Dixon L. (2024). The global and national energy systems techno-economic (GNESTE) database: Cost and performance data for electricity generation and storage technologies. Data Brief.

[6] Göpfert J., Kuckertz P., Weinand J. (2022). Findings of the Association for Computational Linguistics: EMNLP 2022.

[7] Vaswani A., Shazeer N., Parmar N. (2017). Advances in Neural Information Processing Systems.

[8] Devlin J., Chang M.-W., Lee K. (2019). Proceedings of the 2019 Conference of the North American Chapter of the Association for Computational Linguistics: Human Language Technologies, Volume 1 (Long and Short Papers).

[9] Lafferty J.D., McCallum A., Pereira F.C.N. (2001). Proceedings of the Eighteenth International Conference on Machine Learning, ICML ’01.

[10] Huang Z., Xu W., Yu K. (2015). Bidirectional LSTM-CRF Models for Sequence Tagging. arXiv.

[11] Foppiano L., Romary L., Ishii M. (2019). Proceedings of the ACM Symposium on Document Engineering 2019.

[12] Gangwar A., Jain S., Sourav S. (2021). Proceedings of the 15th International Workshop on Semantic Evaluation (SemEval-2021).

[13] Karia N., Kaushal A., Mallick F. (2021). Proceedings of the 15th International Workshop on Semantic Evaluation (SemEval-2021).

[14] Therien B., Bagherzadeh P., Bergler S. (2021). Proceedings of the 15th International Workshop on Semantic Evaluation (SemEval-2021).

[15] Davletov A., Gordeev D., Arefyev N. (2021). Proceedings of the 15th International Workshop on Semantic Evaluation (SemEval-2021).

[16] Avram A.-M., Zaharia G.-E., Cercel D.-C. (2021). Proceedings of the 15th International Workshop on Semantic Evaluation (SemEval-2021).

[17] Hoffmann R., Zhang C., Weld D.S. (2010). Proceedings of the 48th Annual Meeting of the Association for Computational Linguistics.

[18] Vlachos A., Riedel S. (2015). Proceedings of the 2015 Conference on Empirical Methods in Natural Language Processing.

[19] Madaan A., Mittal A., Mausam (2016). Proceedings of the Thirtieth AAAI Conference on Artificial Intelligence, AAAI’16.

[20] Mykowiecka A., Marciniak M., Kupść A. (2009). Rule-based information extraction from patients’ clinical data. J. Biomed. Inform..

[21] Zhang J., El-Gohary N.M. (2016). Semantic NLP-Based Information Extraction from Construction Regulatory Documents for Automated Compliance Checking. J. Comput. Civ. Eng..

[22] Lamm M., Chaganty A., Manning C.D. (2018). Proceedings of the 2018 Conference on Empirical Methods in Natural Language Processing.

[23] Friedrich A. (2020). Proceedings of the 58th Annual Meeting of the Association for Computational Linguistics.

[24] Intxaurrondo A., Agirre E., Lopez de Lacalle O. (2015). Proceedings of the 2015 Conference of the North American Chapter of the Association for Computational Linguistics: Human Language Technologies.

[25] Saha S., Pal H. (2017). Proceedings of the 55th Annual Meeting of the Association for Computational Linguistics (Volume 2: Short Papers).

[26] Kohler C., Jr R.D. (2021). What’s in a Measurement? Using GPT-3 on SemEval 2021 Task 8 - MeasEval. arXiv.

[27] Sipilä M., Mehryary F., Pyysalo S. (2025). Question Answering models for information extraction from perovskite materials science literature. Commun. Mater..

[28] Ray S. (2011).

[29] Ramshaw L., Marcus M. (1995). Third Workshop on Very Large Corpora.

[30] Li X., Chan S., Zhu X., Wang M., Zitouni I. (2023). Proceedings of the 2023 Conference on Empirical Methods in Natural Language Processing: Industry Track.

[31] Cheung J., Zhuang Y., Li Y., Duh K., Gomez H., Bethard S. (2024). Proceedings of the 2024 Conference of the North American Chapter of the Association for Computational Linguistics: Human Language Technologies (Volume 1: Long Papers).

[32] Foppiano L., Lambard G., Amagasa T. (2024). Mining experimental data from materials science literature with large language models: An evaluation study. Sci. Technol. Adv. Mater.: Methods.

[33] Lu Y.-T., Huo Y., Chen C.-C. (2025). Proceedings of the Joint Workshop of the 9th Financial Technology and Natural Language Processing (FinNLP), the 6th Financial Narrative Processing (FNP), and the 1st Workshop on Large Language Models for Finance and Legal (LLMFinLegal).

[34] Wei Z., Su J., Wang Y., Jurafsky D., Chai J., Schluter N., Tetreault J. (2020). Proceedings of the 58th Annual Meeting of the Association for Computational Linguistics.

[35] Li X., Yin F., Sun Z., Korhonen A., Traum D., Màrquez L. (2019). Proceedings of the 57th Annual Meeting of the Association for Computational Linguistics.

[36] Harper C., Cox J., Kohler C. (2021). Proceedings of the 15th International Workshop on Semantic Evaluation (SemEval-2021).

[37] Schrader T.P., Finco M., Grünewald S., Ghosal T. (2023). Proceedings of the Second Workshop on Information Extraction from Scientific Publications.

[38] Vrandečić D., Krötzsch M. (2014). Wikidata: a free collaborative knowledgebase. Commun. ACM.

[39] Wikimedia Foundation (2001). Wikipedia, the free encyclopedia. https://en.wikipedia.org.

[40] Göpfert J., Kuckertz P., Weinand J.M. (2025). Wiki-Quantities and Wiki-Measurements: Datasets of quantities and their measurement context from Wikipedia. Sci. Data.

[41] Mysore S., Jensen Z., Kim E., Friedrich A., Zeyrek D., Hoek J. (2019). Proceedings of the 13th Linguistic Annotation Workshop.

[42] Li Y., Martschat S., Ponzetto S.P., Demner-fushman D., Ananiadou S., Cohen K. (2023). The 22nd Workshop on Biomedical Natural Language Processing and BioNLP Shared Tasks.

[43] Shamsabadi M., D’Souza J., Auer S., Graham Y., Purver M. (2024). Findings of the Association for Computational Linguistics: EACL 2024.

[44] Sun Z., Li J., Pergola G., Goldberg Y., Kozareva Z., Zhang Y. (2022). Proceedings of the 2022 Conference on Empirical Methods in Natural Language Processing.

[45] Foppiano L., Dieb S., Suzuki A. (2021). SuperMat: construction of a linked annotated dataset from superconductors-related publications. Sci. Technol. Adv. Mater.: Methods.

[46] Müller G., Kullmann F., Linssen J. (2025). The costs of future energy technologies: A comprehensive review of power-to-X processes. J. CO2 Util..

[47] Thawani A., Pujara J., Ilievski F. (2021). Proceedings of the 2021 Conference on Empirical Methods in Natural Language Processing.

[48] Wolf T., Debut L., Sanh V., Liu Q., Schlangen D. (2020). Proceedings of the 2020 Conference on Empirical Methods in Natural Language Processing: System Demonstrations.

[49] Ansel J., Yang E., He H. (2024). Proceedings of the 29th ACM International Conference on Architectural Support for Programming Languages and Operating Systems.

[50] Mölder F., Jablonski K.P., Letcher B. (2021). Sustainable data analysis with Snakemake. Tech. Rep..

[51] Nakayama H. (2018). seqeval: A Python framework for sequence labeling evaluation. https://github.com/chakki-works/seqeval.

[52] Rajpurkar P., Jia R., Liang P., Gurevych I., Miyao Y. (2018). Proceedings of the 56th Annual Meeting of the Association for Computational Linguistics (Volume 2: Short Papers).

[53] Hugging Face (2020). Evaluate: A library for easily evaluating machine learning models and datasets. https://github.com/huggingface/evaluate.

[54] Akiba T., Sano S., Yanase T. (2019). Proceedings of the 25th ACM SIGKDD International Conference on Knowledge Discovery & Data Mining, KDD ’19.

[55] Bergstra J., Bardenet R., Bengio Y. (2011). Advances in Neural Information Processing Systems, Vol. 24.

[56] Bhattacharjee B., Trivedi A., Muraoka M., Dernoncourt F., Preoţiuc-Pietro D., Shimorina A. (2024). Proceedings of the 2024 Conference on Empirical Methods in Natural Language Processing: Industry Track.

[57] Gururangan S., Marasović A., Swayamdipta S., Jurafsky D., Chai J., Schluter N., Tetreault J. (2020). Proceedings of the 58th Annual Meeting of the Association for Computational Linguistics.

[58] Webersinke N., Kraus M., Bingler J.A. (2022). ClimateBert: A Pretrained Language Model for Climate-Related Text. arXiv.

[59] Chung H.W., Hou L., Longpre S. (2024). Scaling Instruction-Finetuned Language Models. J. Mach. Learn. Res..

[60] Almasian S., Kazakova V., Göldner P., Bouamor H., Pino J., Bali K. (2023). Proceedings of the 2023 Conference on Empirical Methods in Natural Language Processing.

[61] Liu Y., Ott M., Goyal N. (2019). RoBERTa: A Robustly Optimized BERT Pretraining Approach. arXiv.

[62] Raffel C., Shazeer N., Roberts A. (2020). Exploring the Limits of Transfer Learning with a Unified Text-to-Text Transformer. J. Mach. Learn. Res..

[63] Loshchilov I., Hutter F. (2018). International Conference on Learning Representations: ICLR 2019.

[64] Shazeer N., Stern M. (2018). Proceedings of the 35th International Conference on Machine Learning.

[65] Rajpurkar P., Zhang J., Lopyrev K., Su J., Duh K., Carreras X. (2016). Proceedings of the 2016 Conference on Empirical Methods in Natural Language Processing.

[66] Busch T., Groß T., Linßen J. (2023). The role of liquid hydrogen in integrated energy systems–A case study for Germany. Int. J. Hydrogen Energy.

[67] Haney M.S., Pálovics R., Munson C.N. (2024). APOE4/4 is linked to damaging lipid droplets in Alzheimer’s disease microglia. Nature.

[68] Abu-Shawareb H., Acree R., Adams P. (2024). Achievement of Target Gain Larger than Unity in an Inertial Fusion Experiment. Phys. Rev. Lett..

[69] Walter J.R., Xu S., Rogers J.A. (2024). From lab to life: How wearable devices can improve health equity. Nat. Commun..

[70] Grobid (2008–2025). https://github.com/kermitt2/grobid.

[71] Lopez P., Agosti M., Borbinha J., Kapidakis S., Papatheodorou C., Tsakonas G. (2009). Research and Advanced Technology for Digital Libraries, Lecture Notes in Computer Science.

[72] IRENA. Renewable Power Generation Costs in 2023 (2024). Tech. Rep. International Renewable Energy Agency, Abu Dhabi. https://www.irena.org/-/media/Files/IRENA/Agency/Publication/2024/Sep/IRENA_Renewable_power_generation_costs_in_2023.pdf.

[73] Team, Q (2025). Qwen3 technical report.

[74] Wyss M., Speiser M., Tolis S. (2023). Earthquake fatalities and potency. Nat. Hazards.

[75] Vasconcelos H., Jörke M., Grunde-McLaughlin M. (2023). Explanations Can Reduce Overreliance on AI Systems During Decision-Making. Proc. ACM Hum.-Comput. Interact..

[76] Perry N., Srivastava M., Kumar D. (2023). Proceedings of the 2023 ACM SIGSAC Conference on Computer and Communications Security, CCS ’23.

[77] Dodge J., Ilharco G., Schwartz R. (2020). Fine-Tuning Pretrained Language Models: Weight Initializations, Data Orders, and Early Stopping. arXiv.

