# Supplementary material for: Quinex: Quantitative information extraction from text using open and lightweight LLMs
Source: Innovation (Camb). 2026 Apr 15;7(9):101391. doi: 10.1016/j.xinn.2026.101391 (PMC13556337; doi:10.1016/j.xinn.2026.101391)
Supplement: Supplementary file 1 [file mmc1.pdf]

The Innovation, Volume *7*

Supplemental Information

Quinex: Quantitative information extraction from text using open and lightweight LLMs

Jan Göpfert, Patrick Kuckertz, Gian Müller, Luna Lütz, Celine Körner, Hang Khuat, Detlef Stolten, and Jann Michael Weinand

Contents

S1 Supplemental framework details
- S1.1 Task definitions
- S1.2 Quantity span normalization
- S1.3 Text chunking
- S1.4 Throughput optimization
- S1.5 Flow diagram of the framework

S2 Dataset statistics
- S2.1 Quantity span identification
- S2.2 Measurement context extraction

S3 Detailed results
- S3.1 Quantity span identification
  - S3.1.1 Main results
  - S3.1.2 Supplemental preliminary experiments
- S3.2 Measurement context extraction
  - S3.2.1 Main results
  - S3.2.2 Supplemental preliminary experiments
- S3.3 Quantity span normalization

S4 Curation frontend and annotation format

S5 Question templates
- S5.1 Tested question templates (property-first)
- S5.2 Tested question templates (entity-first)

S6 Tested enclosures

S7 Search queries for application experiments

References

List of Figures

S1 Flow diagram of the Quinex pipeline

S2 Randomly masking tokens in the quantity span identification datasets

S3 Comparing question templates, question orders, and tags to highlight previous answers in context

S4 Frontend for visualization and curation of Quinex predictions

S5 Annotation format

List of Tables

S1 Dataset statistics for quantity span identification

S2 Top 25 most frequent units per quantity span identification dataset

S3 Dataset statistics for measurement context extraction

S4 Top 10 most frequent entities, properties, units, and temporal scope per measurement context extraction dataset

S5 As-reported F1 scores of different quantitative information extraction systems

S6 Final results for quantity span identification

S7 Manual evaluation of the best checkpoint considering alternative correct answers

S8 Initial comparison of base models for quantity span identification

S9 Initial comparison of dataset compositions and orders

S10 Pre-training on a general NER dataset for quantity span identification

S11 Randomly masking tokens in the quantity span identification datasets

S12 Final results for measurement context extraction

S13 Parameter variation of training setup for measurement context extraction

S14 Evaluation results of the quantity parser

S15 Failure modes of the quantity parser

S16 Final question templates

S1 Supplemental framework details

The main article describes the key aspects of the framework in detail. This supplementary section provides additional information on the task description (Section S1.1), the rule-based quantity normalization (Section S1.2), the text chunking approach (Section S1.3), and throughput optimization (Section S1.4). Figure S1 illustrates the Quinex pipeline with a flow diagram.

S1.1 Task definitions

In quantity span identification all *quantities* in a given text are identified. This also includes relative quantities (e.g., in "increased by 5 watt"), imprecise quantities (e.g., in "a few watts"), non-physical quantities (e.g., "five households"), and constants (e.g., "room temperature", "standard atmospheric pressure", or "light speed"). However, due to the approach used to create the Wikipedia-based data, which accounts for most of the data, we do not include quantity modifiers in the quantity spans. Temporal expressions are considered a quantity if they denote a duration (e.g., "5 milliseconds"), but not if they denote a point of time (e.g., "Feb 28th, 2024"). For intervals, lists, or multidimensional quantities, either each individual quantity can be annotated or they can be combined into a single annotation. Splitting an annotation into individual quantities is preferred when the context contains information that applies to some, but not all, of the quantities, as this allows the individual context to be associated with the respective quantities in the measurement context extraction step. For example, intervals such as "from ... to ... " should be split into two quantities if this allows referencing the start and end condition (e.g., individually annotating the quantities in *"For UK specification cars, this increased power from [45 kW]$_{Quantity}$ to [52 kW]$_{Quantity}$."* allows us to link 'UK specification" only to the latter quantity). If none of the individual quantities of a composed quantity are individually contextualized, the composed quantity is preferred to be annotated as a single quantity to reduce the number of quantities for which the more compute-intensive measurement context extraction step is performed.

Measurement context extraction involves extracting the measured entity and property for a given quantity, as well as the qualifiers for the quantitative claim made. Qualifiers include the temporal scope, spatial scope, references, methods, and other qualifiers. Properties can either be annotated within the text or specified separately as free text to account for implicit properties. Annotations should be as expressive as possible. Therefore, entity spans should cover adjacent aliases (e.g., *"[Piz Chavalatsch (also known as "Monte Cavallaccio")]$_{Entity}$"*). Similarly, in *"From a Technology Readiness Level perspective (TRL), the latter process is most advanced with a TRL of [6 to 7]$_{Quantity}$ at least."*, the correct property is 'Technology Readiness Level (TRL)' and not 'TRL'. Annotations can overlap, e.g., in *"According to the 2010 census, Venice has a total area of..."*, the temporal scope is '2010', the method is 'census', and the reference is '2010 census'. Also, alternative and discontinuous entity and qualifier spans should be annotated. Further specifics are evident from the curated data itself.

S1.2 Quantity span normalization

Quantity span normalization is performed using a rule-based parser that breaks down the quantity span into numeric values, units, modifiers, and uncertainty expressions. The parser determines the type of quantity span (single quantity, range, list, ratio, multidimensional, or unknown) and normalizes the values, units, modifiers, and uncertainty expressions. The code is published at `https://github.com/FZJ-IEK3-VSA/quinex-utils`.

The parser expects the quantity span including adjacent quantity modifiers as input. The given quantity span is pre-processed by removing double whitespace, normalizing dashes and mathematical operators, such as equal or greater than, multiplication and division symbols, changing '10 3' and '10^3' to '10^3', and special Unicode number characters such as '¼' to '1⁄4'. Next, the quantity span is tokenized using regular expressions. Known quantity modifier phrases, imprecise quantities, number words, and uncertainty expressions are protected from being split. The tokens are assigned classes (i.e., whitespace, year, number, mathematical operator, range separator, list separator, multidimensional quantity separator, unit, prefixed quantity modifier, and suffixed quantity modifier). Because, only based on the token's surface form, more than one class may be valid for a token, we create a permutation of all reasonable options. Based on the classes, we split the quantity span into individual quantities, and each individual quantity into a value, units, and modifiers for each class sequence in the permutation. Next, the most likely superstructure is chosen by removing duplicates, removing known false positives, and discarding superstructures that could not be successfully segmented. If there are still multiple options, the most simple one, that is, the one with the least quantities, is chosen and a warning is printed. Based on the separators present, we classify the quantity span as a single quantity, range, list, multidimensional, or unknown type.

Having arrived at a single interpretation, each part of each identified quantity is normalized. If this fails for any part of a quantity or a quantity could not be divided into meaningful parts based on the token classes in the first place, another approach is tried: Using normalizers for prefixed modifier, prefixed unit, value, uncertainty expressions pre unit, suffixed unit, uncertainty expressions post unit, and suffixed modifier, in this order, beginning at the first token, the first normalizer is applied to the largest consecutive token sequence until normalization fails. Then the next normalizer starts from there and so on. Quantity modifiers are normalized using a dictionary of known modifier phrases and their normalized form (e.g., "at least" is normalized

to '⩾' and "above" to '>'). Values are interpreted as a numeric data type by first trying to cast them as integers, if that fails, as floats, then as number words, combination of digits and number words, and so forth. Additionally, the order of magnitude and whether the value is imprecise is determined using rules and patterns. Uncertainty expressions are normalized into their lower and upper bounds, unit, and type (i.e., tolerance, confidence interval, or standard deviation).

Units are normalized by linking them to their corresponding QUDT unit class and determining the exponent. The aim is not to divide the compound units into their smallest parts, but to return as few parts as possible, ideally a single QUDT class is equivalent to the given unit. If this is not the case, the unit is likely a compound unit and is split at whitespace, '/', '*', '^', and 4-digit numbers (likely years) to link each part individually to a QUDT unit whilst keeping track of the exponent. Linking to the QUDT ontology is done based on dictionaries of labels and symbols for QUDT units, compiled by combining information from the QUDT[1], OM-2[2], CQE[3] or quantulum3[4], and Wikidata[5]. If the unit is a currency, we check if a year is given after it (e.g., "0.5 $2021/kWh"). Unit parsing is successful if all parts of the unit string have been used exactly once. Ambiguities are currently resolved using hard-coded unit priorities. Next, we try to find a single QUDT unit class that is equivalent to the individual normalized compound unit parts using dimensional analysis. In case of multiple options, the one with lowest Levenshtein distance is chosen. Rules are used to resolve ellipses of units and orders of magnitude (e.g., in "1, 2, 3, and 4 million km" the units and orders of magnitude for the first three quantities are ellipsed). Having linked units to the QUDT and determined the exponents, unit conversion for quantities is possible. Furthermore, the quantity kind (e.g., length, power, etc.) can be determined based on the relations in the QUDT.

Finally, the parser output is classified as likely correct or incorrect by checking if the result is formally valid (e.g., all tokens must have been assigned a role, intervals must have two values, lists at least two, etc.) and by rating the likeliness of the result using heuristics. For example, if all values and units are normalized to numeric data types and QUDT units, the result is more likely correct. Conversely, if an interval has different units for the lower and upper bound or the interval goes from large to small values, the result is more likely incorrect.

S1.3 Text chunking

The number of tokens a transformer model can process in one pass is limited. The models used in this study are constrained to 512 tokens. For the training, development, and test data, it is ensured that each example is no longer than 512 tokens. During inference, however, text must be split into chunks of processable length. For identifying quantities, the text is split in roughly evenly sized chunks (see Figure S1). For this, we adapted the Python library semchunk[6]. For measurement context extraction, each chunk is constructed by taking the largest meaningful chunk that contains the quantity span and iteratively removing or adding parts until it is below the chunk size, going from larger to smaller semantically meaningful chunks. The boundaries of paragraphs, sentences, clauses, and tokens are used to maintain meaningful chunks of text. As information before the quantity span is often more important, text is removed from the back first. If the chunk is still over the chunk size, text is removed from the front. The text chunker is published at `https://github.com/FZJ-IEK3-VSA/llm-chunker`.

S1.4 Throughput optimization

The inference pipeline uses both parallelization and concurrency. First, the text is divided into roughly equally sized chunks that are distributed into batches that fit the GPU's memory and processed in parallel on one or more GPUs. As soon as enough quantities are extracted to fill a batch for measurement context extraction and statement type classification, both tasks are performed in parallel (see Figure S1). Also, if multiple quantity batches are ready at once, they are processed in parallel if enough resources are available. The number of GPUs, parallel models, and batch sizes to use must be optimized individually depending on available hardware and preferences.

S1.5 Flow diagram of the framework

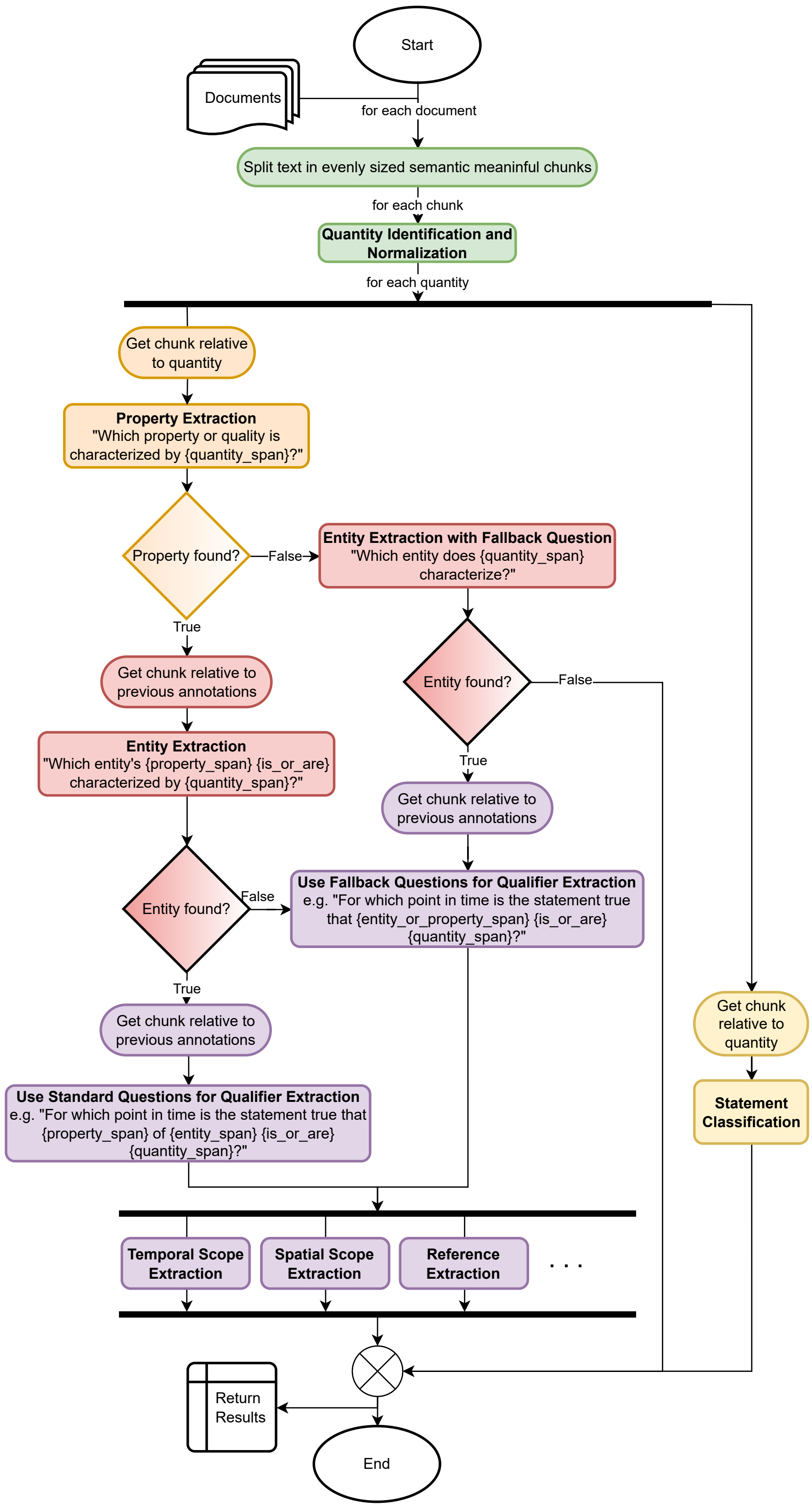

Figure S1. Flow diagram of the Quinex pipeline.

S2 Dataset statistics

This section provides statistics on the datasets created for quantity span identification and measurement context extraction.

S2.1 Quantity span identification

Table S1. Datasets created for quantity span identification and their creation methods, as well as their number of examples, curated examples, and quantities.

Dataset	Creation Method	#Examples	#Curated examples	#Quantities
Custom data	Manually labeled	1 197	100	293
Wiki-Quantities (large)	Programmatically	561 393	289	779 943
Wiki-Quantities (small)	Programmatically	44 729	289	59 504
Wiki-Quantities (tiny)	Programmatically	20 148	289	26 076
Grobid-quantities (Quinex version)	Re-labeled	1312	125	3 784
SOFC-Exp (Quinex version)	Re-labeled	558	558	1 579
MeasEval (Quinex version)	Re-labeled	468	105	1 795
All datasets*	—	48 264	1 177	66 955

*Excluding Wiki-Quantities in large and tiny variant

Table S2. Top 25 most frequent units per quantity span identification dataset. The percentage of all units in the respective dataset is given in parentheses next to the unit.

	SOFC-Exp (Quinex version)	MeasEval (Quinex version)	Grobid-quantities (Quinex version)	Wiki-Quantities (small)	Custom data	All datasets
1	°C (30.3)	% (18.33)	% (19.86)	kW (3.24)	% (14.55)	°C (3.12)
2	V (6.82)	m (5.56)	H (3.61)	km/h (2.71)	s (3.29)	kW (2.94)
3	% (5.62)	K (4.41)	years (3.43)	km (2.67)	ml (3.29)	km (2.55)
4	Ω cm2 (4.21)	mm (3.38)	Hz (2.70)	hp (2.63)	years (2.82)	m (2.49)
5	mW cm-2 (3.95)	°C (3.38)	AU (2.24)	m (2.57)	m (2.82)	km/h (2.46)
6	h (3.88)	ppm (2.23)	km (2.03)	°C (2.55)	min (2.35)	hp (2.38)
7	mW/cm2 (3.28)	μm (2.23)	patients (1.93)	kg (2.50)	h (2.35)	kg (2.33)
8	μm (3.08)	ms (2.11)	μ m (1.86)	acres (2.48)	ppm (2.35)	acres (2.25)
9	W cm-2 (2.68)	kg (1.98)	GHz (1.75)	lb (2.47)	minutes (1.88)	lb (2.24)
10	S cm-1 (2.47)	nm (1.66)	Iron (1.26)	mph (2.44)	patients (1.88)	mm (2.23)
11	Ω·cm2 (2.41)	cm (1.60)	min (1.23)	°F (2.38)	cm (1.88)	mph (2.21)
12	RedOx cycles (1.67)	s (1.53)	kg/mm2 (1.23)	ft (2.32)	mm (1.88)	°F (2.16)
13	A cm-2 (1.54)	‰ (1.47)	mM (1.12)	mm (2.30)	μ m (1.88)	ft (2.11)
14	oC (1.54)	years (1.28)	months (1.09)	mi (2.27)	mL (1.88)	cm (2.06)
15	S·cm-1 (1.27)	days (1.28)	days (1.09)	ha (2.19)	sources (1.41)	mi (2.05)
16	W/cm2 (1.07)	km (1.28)	mm (1.05)	cm (2.19)	km/wk (1.41)	ha (1.99)
17	A/cm2 (1.00)	D (1.09)	hours (1.05)	m2 (2.18)	g/kg/d (1.41)	m2 (1.99)
18	nm (1.00)	Rp (1.09)	° (0.88)	km2 (2.15)	μM (0.94)	km2 (1.95)
19	mA cm-2 (0.94)	h (0.96)	ml (0.84)	in (2.14)	meV/atom (0.94)	in (1.94)
20	S/cm (0.87)	months (0.89)	nm (0.81)	metres (2.07)	cM (0.94)	metres (1.88)
21	Ωcm2 (0.87)	min (0.83)	°C (0.77)	kilometres (2.06)	mV (0.94)	kilometres (1.87)
22	min (0.87)	m s-1 (0.77)	AT/cm (0.74)	miles (2.05)	km (0.94)	miles (1.86)
23	mV (0.80)	MPa (0.70)	pH (0.74)	feet (2.04)	mg/ml (0.94)	feet (1.85)
24	mW·cm-2 (0.80)	keV (0.70)	μ M (0.74)	inches (1.90)	mg/kg (0.94)	inches (1.72)
25	mA/cm2 (0.80)	wt.% (0.70)	m s -1 (0.63)	long tons (1.70)	M (0.94)	long tons (1.54)

S2.2 Measurement context extraction

Table S3. Datasets used for measurement context extraction. As each example is a text with a single annotated quantitative statement, the number of examples, entity annotations, property annotations, and quantity annotations are all equal. A subset of each dataset has been manually curated and marked as correct.

Dataset	Creation Method	#Examples (= #Entity = #Property = #Quantity annotations)	#Examples with entity, property and quantity curated	#Examples with qualifiers also curated	#Qualifier annotations
Quinex-Hydrogen-TechData	Manually labeled	866	731	671	1436
Wiki-Measurements (large)	Programmatically	38738	237	214	3254
Wiki-Measurements (small)	Programmatically	21854	237	214	1499
PolyIE (Quinex version)	Re-labeled	3019	75	70	543
MSP (Quinex version)	Re-labeled	1643	622	29	41
MuLMS (Quinex version)	Re-labeled	1625	410	281	228
MeasEval (Quinex version)	Re-labeled	1367	115	80	430
orkg-R0 (Quinex version)	Re-labeled	759	25	25	2467
PHEE (Quinex version)	Re-labeled	464	50	35	591
SuperMat (Quinex version)	Re-labeled	130	130	119	203
All datasets*	—	48613	2396	1524	9193

*Excluding Wiki-Measurements in small variant

Table S4. Top 10 most frequent entities, properties, units, and temporal scope per measurement context extraction dataset. The case is ignored except for units.

	Entities	Properties	Units	Temporal Scopes
		**Quinex-Hydrogen-TechData**		
1	pem (1.79)	technology readiness... (3.39)	% (18.79)	in 2050 (6.90)
2	hydrogen liquefactio... (1.55)	sec (3.39)	bar (4.92)	currently (5.91)
3	soec (1.31)	cost (3.00)	€/kW (4.29)	current (5.91)
4	methanation (1.31)	investment costs (2.74)	years (3.91)	by 2030 (4.93)
5	biological methanation (1.19)	efficiency (2.74)	$ (3.15)	today (4.43)
6	high-temperature co2... (1.07)	capex (2.35)	kWh/kgLH2 (2.65)	2050 (2.96)
7	pem electrolyser (1.07)	lifetime (1.96)	V (1.89)	in 2030 (2.96)
8	pem electrolyzer (0.95)	exe (1.96)	○C (1.77)	2030 (2.96)
9	pem electrolysers (0.95)	costs (1.83)	€/kW el (1.77)	in 2017 (2.46)
10	pem systems (0.95)	energy efficiency (1.69)	kW (1.64)	by 2050 (2.46)
		**Wiki-Measurements** (small)		
1	it (1.43)	length (20.05)	km (7.40)	2010 (16.48)
2	its (0.27)	area (9.55)	km2 (5.85)	2011 (11.95)
3	coin (0.18)	number of episodes (6.90)	m (5.66)	2020 (6.87)
4	coins (0.09)	number of stories (5.06)	mile (4.38)	2017 (5.49)
5	note (0.08)	population (3.12)	episodes (4.09)	2018 (5.08)
6	lent (0.08)	number of seats (2.98)	metres (3.83)	2019 (4.40)
7	sata (0.07)	elevation (2.47)	$ (3.02)	2016 (3.71)
8	notes (0.07)	total area (2.46)	story (2.74)	2014 (3.43)
9	three-dollar piece (0.06)	number of beds (1.75)	ft (2.55)	2015 (2.88)
10	the amazon (0.05)	height (1.61)	sq mi (2.45)	2021 (1.79)
		**MeasEval** (Quinex version)		
1	beach (1.79)	temperature (2.19)	% (18.58)	in 2010 (50.0)
2	samples (1.33)	depth (1.84)	m (5.89)	by the end of its life (25.0)
3	soil (1.25)	age (1.58)	K (5.48)	after nine weeks in ... (25.0)
4	saturn (0.94)	n (1.40)	mm (4.26)	—

5	cells (0.78)	p-value (1.23)	°C (3.76)	—
6	scale bar (0.62)	thickness (0.88)	ppm (2.45)	—
7	100 mw wind farm (0.62)	concentrations (0.70)	ms (2.45)	—
8	wind (0.55)	temperatures (0.70)	μm (2.29)	—
9	typical gas giant (h... (0.55)	mean temperature (0.70)	cm (2.05)	—
10	c2 model (0.55)	duration (0.61)	kg (1.96)	—
		**MSP** (Quinex version)		
1	mixture (11.72)	heating temperature (4.47)	degC (18.94)	overnight (66.67)
2	solution (9.51)	drying temperature (4.47)	h (16.81)	after complete disso... (33.33)
3	reaction (4.11)	heating duration (3.16)	g (12.15)	—
4	reactants (1.53)	stirring duration (3.16)	mmol (11.18)	—
5	water (0.92)	drying duration (2.75)	mL (10.08)	—
6	product (0.92)	calcination duration (2.27)	min (4.07)	—
7	products (0.86)	calcination temperature (2.27)	% (2.71)	—
8	sample (0.86)	mole ratio (1.44)	mg (2.00)	—
9	h2o (0.86)	amount of v2o5 (1.38)	ml (1.75)	—
10	suspension (0.86)	purity (1.38)	K (1.42)	—
		**orkg-R0** (Quinex version)		
1	covid-19 (49.74)	basic reproduction n... (99.6)	—	2020 (2.20)
2	sars-cov-2 (7.71)	maximum basic reprod... (0.13)	—	across a 1month period (1.86)
3	coronavirus disease ... (6.56)	overall basic reprod... (0.13)	—	2013-2015 (1.53)
4	novel coronavirus (c... (1.80)	population-specific ... (0.13)	—	during the early out... (1.02)
5	ebola virus disease ... (1.29)	—	—	as of may 11, 2020 (0.85)
6	novel coronavirus di... (1.16)	—	—	during the early epi... (0.68)
7	coronavirus disease ... (1.16)	—	—	june 22, 2021 until ... (0.68)
8	covid19 (1.16)	—	—	21-january-2020 to 2... (0.68)
9	coronavirus disease-... (1.03)	—	—	april 3, 2021 to apr... (0.68)
10	novel coronavirus di... (1.03)	—	—	from 23rd january to... (0.68)
		**PolyIE** (Quinex version)		
1	p1 (2.68)	pce (14.56)	% (31.75)	up to now (100.0)
2	p2 (2.58)	v oc (7.79)	eV (17.47)	—
3	p3 (1.21)	j sc (7.46)	V (14.53)	—
4	random terpolymers (0.82)	ff (5.70)	mA cm^-2 (5.81)	—
5	pqcdtb (0.82)	hole mobilities (2.49)	nm (5.77)	—
6	p3tbdtdtbt (0.78)	homo energy level (2.39)	mA / cm^2 (5.39)	—
7	pzndtdtbo (0.78)	hole mobility (2.39)	°C (2.95)	—
8	dpav-pt (0.75)	pces (2.22)	10^-4 cm^2 V-1 s^-1 (1.85)	—
9	psfdtbt (0.65)	homo energy levels (2.19)	cm^2 V-1 s^-1 (1.52)	—
10	pt-tz-dtffbt (0.62)	lumo energy levels (1.53)	10^-3 cm^2 V-1 s^-1 (1.35)	—
		**PHEE** (Quinex version)		
1	carbamazepine (1.29)	dosage (57.69)	weeks (13.83)	—
2	clozapine (0.86)	number of dosages (13.46)	days (13.61)	—
3	linezolid (0.65)	total dosage (5.77)	months (13.61)	—
4	methotrexate (0.65)	cumulative dosage (3.85)	mg (11.79)	—
5	rifampicin (0.43)	toxic dosage (1.92)	years (6.35)	—
6	amodiaquine (0.43)	daily dosage (1.92)	hours (5.22)	—
7	penicillamine therapy (0.43)	total number of doses (1.92)	mg/day (4.54)	—
8	bismuth subsalicylate (0.43)	duration of continuo... (1.92)	week (1.81)	—
9	intravenous acyclovir (0.43)	number of inhalations (1.92)	mg/m2 (1.59)	—
10	cadmium (0.43)	duration of cycle (1.92)	g (1.59)	—
		**MuLMS** (Quinex version)		
1	cell (1.65)	thickness (2.34)	% (11.4)	after carbonization (33.33)
2	grown epitaxial layers (0.90)	relative humidity (rh) (1.95)	nm (9.04)	after each depositio... (16.67)
3	melt into spherulites (0.68)	current density (1.50)	°C (5.77)	before the measurements (16.67)
4	heat a (0.68)	temperature (1.50)	V (4.59)	prior to the adsorpt... (16.67)
5	measurements (0.60)	ultimate tensile str... (1.17)	MPa (4.52)	at the beginning of ... (16.67)

6	heat b (0.60)	scan rate (0.98)	eV (2.78)	—
7	unmodified epoxy pol... (0.60)	pre-corroding duration (0.78)	μm (2.57)	—
8	cdc layer (0.53)	surface roughness (rms) (0.72)	cm-1 (2.23)	—
9	electrolysis (0.53)	diameter (0.72)	Hz (2.23)	—
10	dlwt (0.53)	average magnitudes o... (0.65)	mm (1.46)	—
		**SuperMat** (Quinex version)		
1	srfe 2 as 2 (3.82)	superconducting crit... (55.56)	K (86.61)	in 1998 (28.57)
2	polycrystalline snsb... (3.05)	maximum superconduct... (5.13)	GPa (8.66)	1998 (14.29)
3	first superconductin... (2.29)	superconducting tran... (3.42)	mK (2.36)	in 1970 (14.29)
4	rusr 2 gdcu 2 o 8 sa... (2.29)	superconducting crit... (2.56)	mJ K -2 mol -1 (0.79)	early 2001 (14.29)
5	free ceobis2 single ... (2.29)	curie temperature (t... (1.71)	mJ mol -1 K -2 (0.79)	in 1994 (14.29)
6	sr 2 vfeaso 3–δ (2.29)	bulk superconducting... (1.71)	Oe (0.79)	in the past two years (14.29)
7	plcco crystals (2.29)	sommerfeld coefficient (1.71)	—	—
8	snsb (2.29)	onset temperature of... (0.85)	—	—
9	g-ti3o5 film (2.29)	superconducting anis... (0.85)	—	—
10	ceirin 5 (2.29)	critical pressure fo... (0.85)	—	—
		**All datasets combined**		
1	covid-19 (1.24)	length (12.67)	km (5.40)	2010 (7.78)
2	it (1.01)	area (6.03)	% (4.92)	2011 (5.77)
3	mixture (0.61)	number of episodes (4.34)	m (4.38)	2020 (4.35)
4	solution (0.50)	number of stories (3.19)	km2 (4.21)	2017 (2.66)
5	p1 (0.26)	basic reproduction n... (3.16)	mile (3.15)	2018 (2.40)
6	p2 (0.25)	population (1.97)	episodes (2.94)	2019 (2.08)
7	reaction (0.23)	number of seats (1.88)	metres (2.75)	2016 (1.75)
8	sars-cov-2 (0.19)	pce (1.84)	$ (2.26)	2014 (1.62)
9	its (0.19)	elevation (1.55)	story (1.97)	2015 (1.49)
10	coronavirus disease ... (0.16)	total area (1.55)	ft (1.83)	2021 (0.91)

S3 Detailed results

The results summarized in the main part are presented in greater detail below. This section is organized into the tasks quantity span identification (S3.1), measurement context extraction (S3.2), and quantity span normalization (S3.3). Table S5 summarizes the performance of Quinex in quantity span identification and measurement context extraction by comparing its F1 scores and features with those reported for other systems.

Table S5. As-reported evaluation results (F1) of different systems for quantitative information extraction. Different task definitions, datasets, and metrics were used; therefore, the scores are only a rough indication of the systems' strengths and weaknesses.

System	Quantity	Property	Entity	Qualifiers	Implicit properties	Sub-categorized qualifiers
Quinex Models v0	**98.08**	**87.32**	**82.49**	**90.47**	True	True
	(SQuAD overlap F1 (macro-av. for qualif.); entity-lev. F1 considering alt. correct answ. for quantities; Quinex test set)					
1st place MeasEval[7,8]	86.1	46.7	43.7	16.3	False	False
	(SQuAD overlap F1; MeasEval test set)					
Li et al. (2023)[9]	76.7	45.0	50.5	—	False	—
	(token-based strict F1; end-to-end; MeasEval+MSP+BM)					
Li et al. (2023)[9]	87.7	43.7	43.2	—	False	—
	(SQuAD overlap F1, MeasEval test set)					
CQE[3]	85.6	76.1†		—	False	False
	(F1; for entity any overlap with groundtruth is considered correct; NewsQuant dataset)					
Grobid-quantities[10]*	70.23	20.89†		—	False	—
	(Soft matching F1; MeasEval dataset; probably without training on MeasEval)					

†It is not distinguished between entities and properties.

*Results from https://grobid-quantities.readthedocs.io/en/latest/evaluation-scores/, Accessed on 2024-10-22

S3.1 Quantity span identification

S3.1.1 Main results

Table S6 shows the final results on the quantity span identification task. For the best model (top row), we manually evaluated the model's errors. In many cases both the model's answer and the ground truth are correct, it is debatable whether it should count as an error or not, or the model is correct and the ground truth is not. Table S7 shows the results for the top model when taking into account alternatively correct answers, ignoring debatable errors, or errors involving answers that are slightly too short or too long but include the numeric value and unit.

Table S6. Results of different encoder-only models trained on the quantity identification task using a 50-trial hyperparameter optimization. Different training strategies (training on all data vs. silver Wiki-Quantities (WQ) and then all other data) and levels of filtering Wiki-Quantities (tiny, small, and large) are compared. Results are averaged over two runs and sorted by F1 score on the test set. The highlighted models are published as quinex-quantity-v0-124M (green), quinex-quantity-v0-82M (yellow), and quinex-quantity-v0-30M (red), respectively.

Base Model	Training datasets and their order	F1 (dev)	F1 (test)	P (test)	R (test)	Acc. (test)	#Params
NASA SMD IBM v0.1[11]	silver WQ (tiny) → all w/o silver WQ	94.55	**96.27**	**96.08**	**96.46**	**99.41**	124M
NASA SMD IBM v0.1[11]	all (small)	95.22	95.96	95.63	96.29	99.28	124M
NASA SMD IBM v0.1[11]	silver WQ (small) → all w/o silver WQ	**95.57**	95.76	95.46	96.06	99.31	124M
BioMed-RoBERTa-base[12]	silver WQ (small) → all w/o silver WQ	94.72	95.57	95.81	95.32	99.28	124M
BioMed-RoBERTa-base[12]	silver WQ (tiny) → all w/o silver WQ	94.49	94.98	93.99	96.00	99.32	124M
BioMed-RoBERTa-base[12]	all (small)	94.89	94.83	94.40	95.26	99.26	124M
distilRoBERTa (base) climate f[13]	silver WQ (small) → all w/o silver WQ	94.74	94.50	93.86	95.15	99.17	82M
distilRoBERTa (base) climate f[13]	all (small)	94.26	94.23	93.44	95.03	99.25	82M
distilRoBERTa (base) climate f[13]	silver WQ (tiny) → all w/o silver WQ	95.28	94.10	93.91	94.29	99.12	82M
distilRoBERTa (base) climate f[13]	silver WQ (large) → all w/o silver WQ	94.82	93.58	92.72	94.46	99.07	82M
NASA SMD IBM distil v0.1[11]	silver WQ (small) → all w/o silver WQ	93.42	93.29	92.97	93.61	99.03	30M
NASA SMD IBM distil v0.1[11]	all (small)	93.69	93.04	91.92	94.18	98.95	30M
NASA SMD IBM distil v0.1[11]	silver WQ (large) → all w/o silver WQ	93.04	92.55	91.57	93.55	99.05	30M
NASA SMD IBM distil v0.1[11]	silver WQ (tiny) → all w/o silver WQ	93.21	92.54	91.66	93.44	99.12	30M

Table S7. Qualitative evaluation of the best model checkpoint from the best setup in Table S6 (top row).

Evaluation strictness	F1 (test)	P (test)	R (test)
Consider alternative correct answers	98.08	98.83	97.35
+ Ignore errors if correct value and unit included in span	98.78	99.53	98.04
+ Ignore errors that are debatable	99.25	99.54	98.96

S3.1.2 Supplemental preliminary experiments

Before settling on the final configuration used for quantity span identification, we experimented with different models (see Table S8), dataset compositions and orders (see Table S9), pre-training on a general NER dataset (see Table S10), and randomly masking tokens (see Table S11 & Figure S2). These experiments are presented below. As they guided decisions on how to train the final models, they are only evaluated on the development sets.

Comparing base models. Table S8 compares various encoder-only transformer models on quantity span identification. The differences between the models are very small for a large part of the models. The top 16 models only differ by less than one absolute F1 point. MSR BiomedBERT-large (334M parameters) achieves the highest F1 score on the development set with 94.32%. The highest F1 score of the medium-sized models (124M parameters) is achieved with BioMed-RoBERTa-base and NASA SMD IBM v0.1, which are only 0.36 and 0.38 absolute F1 points worse, respectively. The best trade-off between model size and performance of the smaller models is given by distilRoBERTa (base) climate f and distilRoBERTa base (82M parameters), which are only 0.83 and 0.84 F1 points worse than the best model, respectively. The even smaller TinyBERT variants performed considerably worse (>4.92 absolute F1 drop). SciBERT consistently outperformed BERT for the same model size. However, the difference between base and domain-adapted RoBERTa- and DistilRoBERTa-based models is marginal. There is no clear indication as to whether cased or uncased variants perform better.

Table S8. To compare different encoder-only transformer models in quantity span identification, we fine-tune them for 6 epochs on all silver data, excluding Wiki-Quantities, and subsequently on all gold data for another 8 epochs. We exclude Wiki-Quantities to save compute resources, as it is large in size compared to the other datasets. We search for the optimal learning rate and batch size in the space of learning rates between 1e-6 and 1e-4 as well as batch sizes of 8 and 16 for 10 trials. We did not choose larger batch sizes as they would not fit on the GPU for some of the models. After the first runs, we exclude all models which are more than 2% worse than the best scoring model in F1 score and repeat the experiment. This time, training for 8 epochs on both datasets using a batch size of 8, 16, and 32 (where possible) and optimizing only the learning rate. For promising model candidates which are close to each other in terms of F1, we repeat the experiment for more random seeds.

Base model	$F1_{mean}$ ($F1_{max}$)	F1 drop	Precision	Recall	Speed*	#Runs
MSR BiomedBERT-large[14]	**94.32** (94.83)	**0.00**	**93.85**	94.80	1.20	6
RoBERTa (large)[15]	94.06 (94.59)	0.28	93.23	**94.90**	1.10	6
XLM-RoBERTa (large)[16]	93.98 (**94.99**)	0.36	93.40	94.56	1.00	6
BioMed-RoBERTa-base[12]	93.98 (94.56)	0.36	93.20	94.77	3.00	10
NASA SMD IBM v0.1[11]	93.96 (94.48)	0.38	93.21	94.73	3.00	10
BERT for Patents[17]	93.89 (94.22)	0.46	93.12	94.67	1.20	6
RoBERTa (base)[15]	93.82 (94.24)	0.53	93.00	94.65	2.90	10
MSR BiomedBERT-base[14]	93.81 (94.28)	0.54	93.04	94.59	3.00	10
Material SciBERT (TPU)[18]	93.76 (94.31)	0.60	93.17	94.35	2.90	7
EnvRoBERTa-base[19]	93.71 (94.1)	0.65	92.92	94.51	2.90	7
GovRoBERTa-base[19]	93.69 (94.18)	0.67	92.81	94.58	2.90	7
SciBERT (uncased)[20]	93.64 (94.02)	0.72	93.10	94.18	3.00	4
XLM-RoBERTa (base)[16]	93.57 (93.86)	0.79	92.75	94.42	2.60	7
SciBERT (cased)[20]	93.55 (93.9)	0.81	93.05	94.06	2.90	7
distilRoBERTa (base) climate f[13]	93.53 (93.98)	0.83	92.39	94.70	4.50	10
DistilRoBERTa (base)[21]	93.53 (94.11)	0.84	92.60	94.48	4.60	10
BioBERT-base (cased) v1.2	93.08 (93.08)	1.32	91.87	94.31	2.70	1
BERT-base (uncased)[22]	93.03 (93.43)	1.36	92.18	93.90	2.80	4
DistilBERT-base (cased)[21]	93.02 (93.02)	1.38	91.76	94.31	4.50	1
Bio+Clinical BERT[23]	92.88 (92.88)	1.53	91.84	93.93	2.70	1
DistilBERT-base (uncased)[21]	92.70 (92.70)	1.72	91.50	93.93	4.30	1
BERT-base (cased)[22]	92.48 (92.48)	1.95	91.68	93.30	2.70	1
MobileBERT[24]	92.37 (92.37)	2.07	91.45	93.30	2.50	1
TinyBioBERT[25]	89.68 (89.68)	4.92	88.25	91.15	5.90	1
TinyBERT (6L, 768D)[26]	88.04 (88.04)	6.66	85.92	90.27	4.50	1
TinyClinicalBERT[27]	87.32 (87.32)	7.42	84.33	90.52	6.80	1
TinyPubMedBERT v1.0[28]	86.34 (86.34)	8.46	83.73	89.13	**7.80**	1
TinyBERT (4L, 312D)[26]	84.77 (84.77)	10.13	80.92	89.00	7.30	1

* Normalized evaluation samples per second

Datasets and their order. Table S9 compares different compositions and sequences of training datasets for quantity span identification. The results indicate that the best performing dataset sequences are 'silver Wiki-Quantities → all w/o silver Wiki-Quantities' (that is, first training on the non-curated Wiki-Quantities subset and subsequently on all other data) and 'all' (that is, direclty training on a mix of all data). Interestingly, distilRoBERTa (base) climate f yielded the third best result, just 0.42 absolute F1 points worse than the best performing model despite being considerably smaller. We also tested longer sequences of fine-tuning steps not shown in the table (e.g., training on three Wiki-Quantities variants of decreasing size for 1 epoch each in succession), but found no benefit.

Table S9. To assess which datasets to use in which order, we fine-tune three different sized encoder-only transformer models on different combinations of training datasets for quantity span identification. The results per model are averaged over two runs with different random seeds, using a batch size of 16 and 20 trials to optimize the learning rate. For each dataset, we train for 8 epochs. The tiny variant of Wiki-Quantities is used.

Model	Training datasets and their order	$F1_{mean}$	Precision	Recall	$F1_{max}$
MSR BiomedBERT-large[14]	silver Wiki-Q. → all w/o silver Wiki-Q.	**94.89**	94.71	**95.07**	**95.14**
MSR BiomedBERT-large[14]	all	94.74	**94.93**	94.56	94.89
distilRoBERTa (base) climate f[13]	silver Wiki-Q. → all w/o silver Wiki-Q.	94.47	93.88	**95.07**	94.47
BioMed-RoBERTa-base[12]	all	94.45	94.21	94.69	94.59
MSR BiomedBERT-large[14]	silver → gold	94.34	93.92	94.75	94.34
distilRoBERTa (base) climate f[13]	all w/o silver Wiki-Q.	94.07	93.45	94.69	94.58
BioMed-RoBERTa-base[12]	all w/o silver Wiki-Q.	94.02	93.61	94.44	94.07
BioMed-RoBERTa-base[12]	silver Wiki-Q. → silver w/o Wiki-Q. → gold	94.02	93.17	94.88	94.31
BioMed-RoBERTa-base[12]	silver Wiki-Q. → all w/o silver Wiki-Q.	93.94	93.38	94.50	94.23
distilRoBERTa (base) climate f[13]	all	93.93	93.49	94.37	94.10
distilRoBERTa (base) climate f[13]	silver → gold	93.85	92.77	94.94	93.87
MSR BiomedBERT-large[14]	silver Wiki-Q. → silver w/o Wiki-Q. → gold	93.80	92.93	94.69	93.84
BioMed-RoBERTa-base[12]	silver → gold	93.80	92.98	94.63	93.97
MSR BiomedBERT-large[14]	all w/o silver Wiki-Q.	93.72	93.58	93.87	93.92
BioMed-RoBERTa-base[12]	silver w/o Wiki-Q. → gold	93.61	92.79	94.44	93.80
distilRoBERTa (base) climate f[13]	silver w/o Wiki-Q. → gold	93.60	92.42	94.82	93.69
MSR BiomedBERT-large[14]	silver w/o Wiki-Q. → gold	93.39	92.98	93.81	93.43
distilRoBERTa (base) climate f[13]	silver Wiki-Q. → silver w/o Wiki-Q. → gold	93.33	92.02	94.69	93.88

all = Training on all data, that is, combine and mix all silver and gold data in a single dataset
all w/o silver Wiki-Q. = Same as above but excluding the silver set of Wiki-Quantities
silver Wiki-Q. → all w/o silver Wiki-Q. = Training first on all silver Wiki-Quantities data before training on all other data for 8 epochs
silver → gold = Training first on all silver data before training on all gold data for 8 epochs
silver w/o Wiki-Q. → gold = Same as above but excluding the silver set of Wiki-Quantities
silver Wiki-Q. → silver w/o Wiki-Q. → gold = Same as above but train on silver Wiki-Quantities first
silver Wiki-Q. → silver → gold = Same as above but including silver Wiki-Quantities in the silver data

Pre-training on a general NER dataset. Table S10 shows the results of training distilRoBERTa (base) climate f on quantity span identification, comparing initial pre-training on general NER datasets with not doing so. The results indicate that pre-training on a general NER dataset slightly worsens performance, but the effect is minimal. Therefore, for the remainder of this study, pre-training on a general NER dataset is not used.

Table S10. To assess whether to train on a general NER dataset first, we fine-tune distilRoBERTa (base) climate f on 'silver Wiki-Quantities → all w/o silver Wiki-Quantities' with and without first training on OntoNotes v5[29]. We map PERCENT, MONEY, and QUANTITY labels to QUANTITY and ignore all other labels in OntoNotes. We do not further modify the dataset. Therefore, the quantity annotations are not in line with the annotation guideline: quantity modifiers such as 'at least' are included in the quantity spans and quantities such as '3 days' are not considered a quantity, as opposed to the rest of the datasets. We use a batch size of 16 and optimize the learning rate with 20 trials for each dataset. Results are averaged over four runs (two random seeds and two Wiki-Quantities variants).

Training datasets and their order (Numbers denote max. epochs, only the best checkpoint is kept)	$F1_{mean}$	Precision	Recall	$F1_{max}$
30x silver Wiki-Q. (small/tiny) → 30x all w/o Wiki-Q.	**94.70**	**94.19**	**95.23**	94.85
5x OntoNotes → 15x silver Wiki-Q. (small/tiny) → 30x all w/o Wiki-Q.	94.57	93.95	95.20	95.02
20x OntoNotes → 30x silver Wiki-Q. (small/tiny) → 30x all w/o Wiki-Q.	94.48	93.97	95.01	**95.05**
1x OntoNotes → 30x silver Wiki-Q. (small/tiny) → 30x all w/o Wiki-Q.	94.39	93.65	95.13	94.47
30x silver Wiki-Q. (small/tiny)	88.55	91.62	85.68	88.73

Randomly masking tokens. We hypothesize that masking tokens improves performance because it forces the LLM to consider the context around a token to determine whether it belongs to a quantity span and prevents it from memorizing typical quantities. Figure S2 shows the F1 scores for DistilRoBERTa-base models trained on all silver data and then all gold data with different token mask probabilities. For each token, the probability to be masked is the same, whether it is part of a quantity span or not. There is a clear decreasing trend in F1 with increasing token mask probability. As one would expect, masking both datasets results in a steeper decline in F1 compared to only masking the first dataset.

Table S11 shows the results for training DistilRoBERTa-base on the dataset sequences 'silver Wiki-Q. → all w/o silver Wiki-Q.' and 'OntoNotes → silver Wiki-Q. → all w/o silver Wiki-Q.' always using hyperparameter optimization. Unlike the negative trends observed in Figure S2, the results do not show a clear trend. Transformer-based language models are very robust with respect to randomly masking tokens as one would expect based on their pre-training objective. As randomly masking tokens does not seem to improve results, we do without it for the rest of the study.

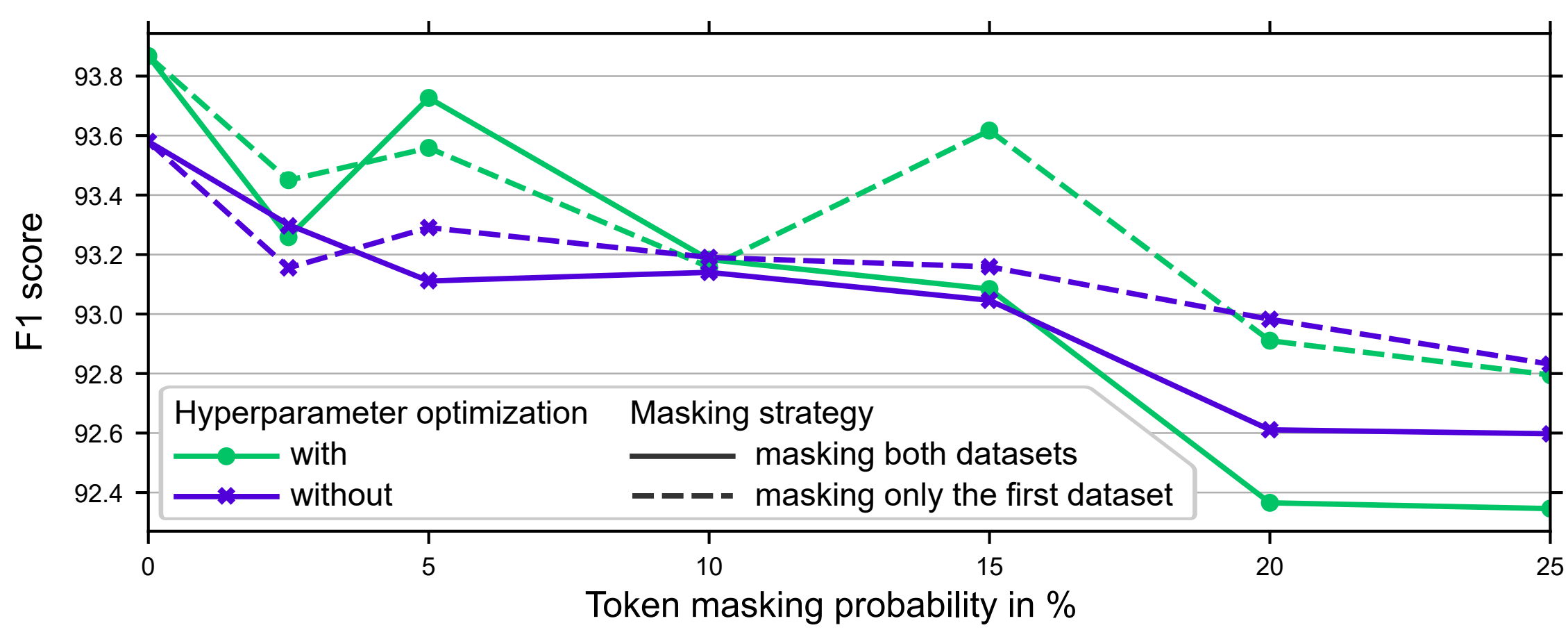

Figure S2. To assess if randomly masking tokens during fine-tuning on the quantity span identification task improves performance, we compare DistilRoBERTa-base models trained with different probabilities for replacing a token with <mask>. For each token, the probability to be masked is the same, whether it is part of a quantity span or not. We fine-tune DistilRoBERTa-base first on all silver data and then on all gold data, using the tiny Wiki-Quantities variant. We compare masking only the silver data to masking both datasets, as well as performing hyperparameter optimization to not performing it. The results are averaged over six runs with different random seeds, except for the results with hyperparameter optimization, where only a single run was performed.

Table S11. To assess if randomly masking tokens during fine-tuning on the quantity span identification task improves performance, we compare DistilRoBERTa-base models trained on 'silver Wiki-Quantities (small) → all w/o silver Wiki-Quantities' or 'OntoNotes → silver Wiki-Quantities (small) → all w/o silver Wiki-Quantities' with different probabilities for replacing a token with <mask>. For each dataset, a 30-trial hyperparameter optimization is performed. Results are averaged over three runs with different seeds.

Token mask probability in % per dataset	F1	Precision	Recall
2.5 → 0	**94.88**	94.15	**95.62**
5 → 5	94.87	**94.63**	95.11
30 → 0 → 0	94.81	94.18	95.45
0 → 0	94.80	94.29	95.32
5 → 0	94.79	94.21	95.36
5 → 0 → 0	94.76	94.21	95.32
10 → 0	94.75	94.40	95.11
15 → 0 → 0	94.61	94.20	95.03
2.5 → 2.5	94.52	94.19	94.86
10 → 0 → 0	94.45	93.91	94.99
0 → 0 → 0	94.17	93.70	94.65
10 → 10 → 0	94.15	93.73	94.56
10 → 10	94.12	93.84	94.40
5 → 5 → 5	94.08	93.40	94.77
10 → 5 → 2.5	94.06	93.76	94.35
20 → 0 → 0	93.90	93.08	94.73
25 → 0 → 0	93.84	93.33	94.35
10 → 5 → 0	93.66	93.39	93.93

S3.2 Measurement context extraction

S3.2.1 Main results

Table S12 shows the final results on the measurement context extraction task. The setup used in the final results is based on preliminary experiments optimizing various variables of the multi-turn question answering approach. They are presented in the following subsection.

Table S12. F1 scores on the test set for FLAN-T5[30] models fine-tuned on measurement context extraction. The models are first trained on all silver data and then trained on all gold data for a maximum of 20 and 35 epochs, respectively. The number of hyperparameter optimization trials per model is set relative to their training time in previous runs. For the first dataset, 42, 15, and 5 trials are used for the small, base, and large model, respectively. For the second dataset, 100, 36, and 12 trials are used for the small, base, and large model, respectively. Overall F1 scores on the development set are 88.26, 86.83, and 85.14 for the large, base, and small model, respectively. The models are published as quinex-context-v0-783M, quinex-context-v0-248M, and quinex-context-v0-77M, respectively.

**Model size** (#Param.; Speed*)	**Overall** *Micro-average*	**Entity and property** *Macro-average*	Property	Entity	**Qualifiers** *Macro-average*	Spatial Scope	Temp. Scope	Method	Reference	Other
Large (783M; 1.0)	88.29	84.91	87.32	82.49	90.47	97.40	94.47	91.02	92.37	77.11
Base (248M; 2.5)	86.31	83.82	85.08	82.56	87.94	97.69	93.52	89.54	89.26	69.67
Small (77M; 5.7)	83.32	80.13	81.68	78.58	85.41	97.40	92.52	86.65	86.41	64.08

*Normalized test samples per second

S3.2.2 Supplemental preliminary experiments

Question order, question templates, and context highlighting. Figure S3 shows the results for optimizing (a) the question order and templates, as well as (b) the symbols and tags to highlight the already identified quantity and property in the context for the subsequent questions.

For Figure S3a, we tested the permutation of the entity and property questions listed in Section S5. The results clearly

show that asking for the property first yields better results than asking for the entity first. The fallback questions, which are used if a previous prediction is empty, are "Which entity is described by $\{quantity\_span\}$?" when asking for the property first and "Which property is described by $\{quantity\_span\}$?" when asking for the entity first, and are not varied, as varying the fallback entity question yielded negligible differences in performance (0.18 F1 points between worst and best result, testing eight fallback questions averaged over six runs with different random seeds). This is not surprising as only a small proportion of examples use the fallback question. Also, asking "What entity..." instead of "Which entity..." in both the normal and the fallback entity question results in negligible differences.

For Figure S3b, we test the permutation of symbols and tags listed in Section S6 for highlighting the quantity and property in the context. The best result (86.93% F1) was achieved by using $...$ to enclose the quantity and **...** to enclose the property. Enclosing the property with double asterisks but not enclosing the quantity was the 171st worst combination out of 1226 tested with 85.91% F1. Neither enclosing the property nor the quantity was the fourth worst with 85.70%. However, the combination that surrounded the quantity with double asterisks without surrounding the property was the 326th best with 86.34 F1. The double asterisks and dollar signs may work best because double asterisks are used in Markdown to make text bold, and dollar signs are used in LaTeX for inline math mode. In both (a) and (b), qualifier questions are not considered.

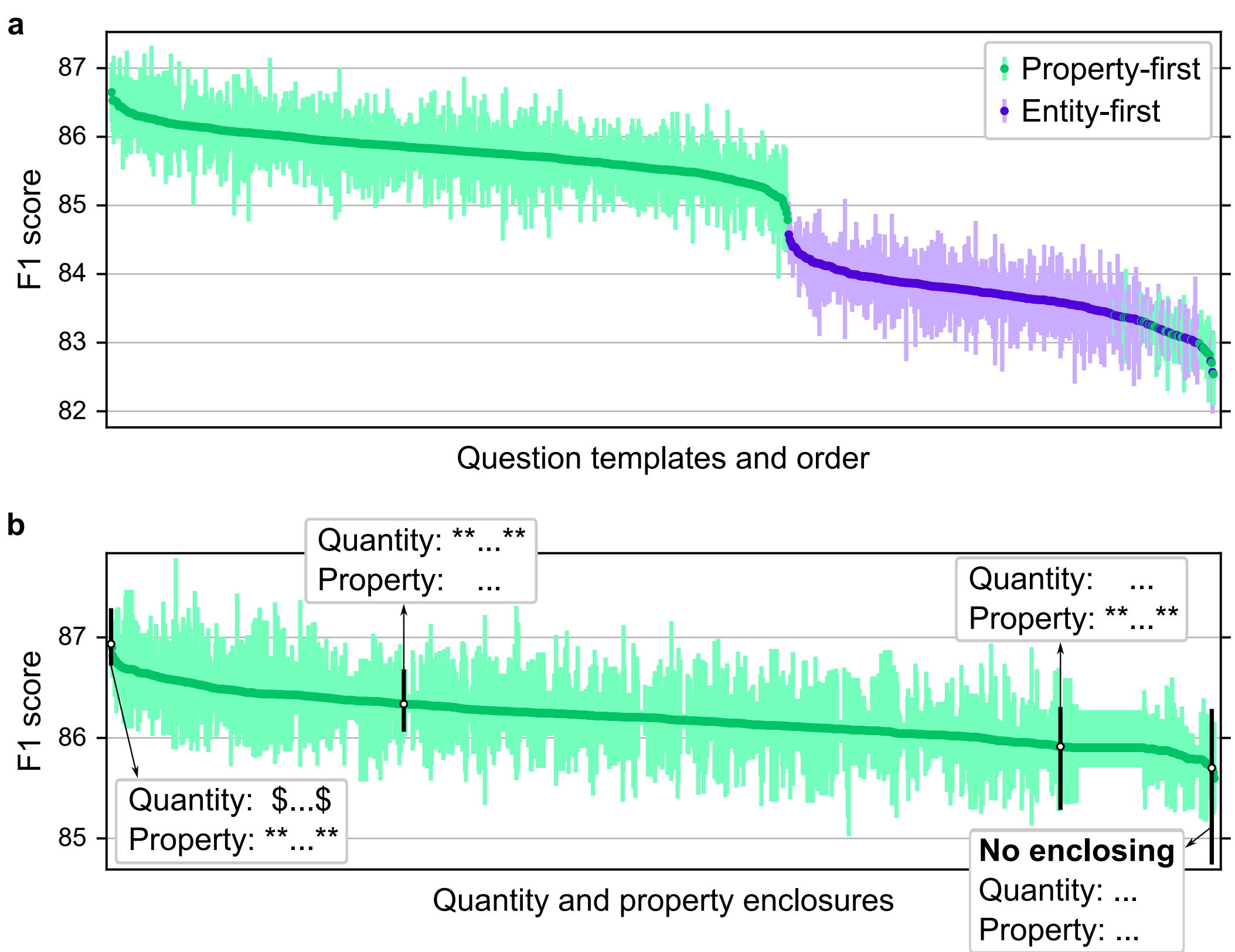

Figure S3. F1 scores for extracting measured properties and entities for fine-tuned FLAN-T5-base models[30] for **(a)** different question templates and orders, and **(b)** different tags to highlight the respective quantity and property in the text. The question order is marked in green for variants that ask for the property first, and in purple for variants that ask for the entity first. In (a), only the quantity span was highlighted in the context for the entity and property questions, using two asterisks to the left and right. In (b), the symbols for highlighting quantities are varied, and for entity questions, the property is also highlighted. All enclosures that are not labeled with a text field enclose both the quantity and property. The results are averaged over three runs with different random seeds on the development set using a learning rate of 1e-04, which performed best among 1e-03, 1e-04, and 1e-05 for both question orders. The error bars show the spread of results using different seeds.

Further parameter variation. Table S13 shows the results of varying additional parameters in training the measurement context extraction models. The results provide insight into which datasets to use, whether to mix gold and silver data, highlighting information in the questions and contexts, filtering Wiki-Measurements, and using a joint model for entity-property and qualifier extraction, and more.

Table S13. Results on the test set for a parameter variation to find an optimal approach for training measurement context extraction models. Note that only the results on the development set guided decisions on how to train the final set of models. The test sets were used only afterwards. Three runs with different random seeds are averaged. For each run a 10-trial hyperparameter optimization was performed. The table is divided by questions, which are answered by comparing rows from one gray row to the next gray row. For each question, the cells for the column of interest are color-coded according to whether they have a positive (green), negative (red), or neutral (yellow) effect.

Datasets and their order (Numbers denote max. epochs, only the best checkpoint is kept)	Joint model	Questions marked	Entity marked	Added context	Wiki-M. filtered	**Overall F1** *micro-average*	**Entity & Property F1** *macro-average*	**All qualifiers F1** *macro-average*
Whether to use silver data or not?								
20x silver → 35x gold	True	False	True	False	True	86.16	83.14	88.13
40x gold	True	False	True	False	True	85.40	80.80	88.36
What sequence of training datasets to use?								
20x silver → 35x gold	True	False	True	False	True	86.16	83.14	88.13
20x all → 35x gold	True	False	True	False	True	86.14	83.48	87.88
20x silv. WM → 35x all w/o silv. WM	True	False	True	False	True	86.12	82.90	88.21
40x all	True	False	True	False	True	85.01	82.36	86.76
Whether to perform pre-training on a general QA dataset or not?								
20x silver → 35x gold	True	False	True	False	True	86.16	83.14	88.13
3x SQuAD → 20x silv. → 35x gold	True	False	True	False	True	85.95	82.62	88.10
Whether to use a joint model or separate ones?								
20x silver → 35x gold	True	True	True	False	True	86.00	83.25	87.79
20x silver → 35x gold	False	True	True	False	True	—	82.68	—
40x gold*	False	True	True	False	True	—	—	87.69
20x silver → 35x gold	True	False	True	False	True	86.16	83.14	88.13
20x silver → 35x gold	False	False	True	False	True	—	83.56	—
40x gold*	False	False	True	False	True	—	—	88.07
Whether to mark previous predictions in the questions?								
20x silver → 35x gold	True	False	True	False	True	86.16	83.14	88.13
20x silver → 35x gold	True	True	True	False	True	86.00	83.25	87.79
20x silver → 35x gold	True	False	False	False	True	86.40	83.87	88.07
20x silver → 35x gold	True	True	False	False	True	86.41	83.76	88.15
Whether to mark entities in the context for qualifier questions?								
20x silver → 35x gold	True	False	True	False	True	86.16	83.14	88.13
20x silver → 35x gold	True	False	False	False	True	86.40	83.87	88.07
20x silver → 35x gold	True	True	True	False	True	86.00	83.25	87.79
20x silver → 35x gold	True	True	False	False	True	86.41	83.76	88.15
Whether to add more context to Wiki-Measurements data or not?								
20x silver → 35x gold	True	False	True	False	True	86.16	83.14	88.13
20x silver → 35x gold	True	False	True	True	True	86.62	83.20	88.82
Whether to filter Wiki-Measurements or not?								
20x silver → 35x gold	True	False	True	False	True	86.16	83.14	88.13
20x silver → 35x gold	True	False	True	False	False	86.53	83.58	88.45
How much does gold data add to performance?								
20x silver → 35x gold	True	False	True	False	True	86.16	83.14	88.13
20x silver	True	False	True	False	True	73.14	77.52	70.51
20x silver WM	True	False	True	False	True	31.61	56.78	15.92
3x SQuAD	True	False	True	False	True	51.55	34.01	62.61

*The silver data contains almost no qualifier annotations, hence it is only trained on the gold data for qualifier-only models

WM=Wiki-Measurements, silver=all silver data, gold=all gold data

S3.3 Quantity span normalization

Table S14 shows the results of the evaluation of the rule-based quantity parser on annotated data from Grobid-quantities[10,31]. We use the first 25% of quantities in ./RESOURCES/DATASET/QUANTITIES/CORPUS to improve the quantity parser and then evaluate on the unseen remaining 75% of data (Corpus 2, unseen). Afterwards, we make simple adjustments to the parser based on the observed errors, and then evaluate it again to establish the upper limit of its performance when adapting it to the corpus at hand (Corpus 2, seen). Using the improved version of the parser, we evaluate it on another unseen ./RESOURCES/DATASET/QUANTITIES/EVALUATION (Corpus 1, unseen). Afterwards, we again modify the parser based on the observed errors and evaluate it again to establish its upper performance limit (Corpus 1, seen). We also evaluate the parser's self-assessment reliability, that is, how reliable is the parser at judging whether its output is correct or not. Table S15 provides statistics on the observed failure modes of the rule-based quantity parser.

Table S14. Accuracy of the rule-based quantity parser on annotated data from Grobid-quantities[10,31], without (unseen) and with (seen) adjusting the parser based on observed errors. Self-assessment reliability describes how well the parser judges whether its output is correct or not.

		Corpus 1		Corpus 2	
		(unseen)	*(seen*)*	*(unseen)*	*(seen*)*
Performance	Failed / total examples	62 / 757	31 / 757	276 / 2756	172 / 2756
	**Accuracy****	**91.93%**	96.03%	**91.24%**	95.07%
Self-assessment reliability***	Precision	97.85%	99.45%	97.59%	98.28%
	Recall	98.42%	100.00%	99.64%	99.69%
	F1	98.13%	99.73%	98.60%	98.98%

* if unseen is False the parser was adapted based on the errors of the first run on the unseen data and evaluated again

** excluding errors of type 'non-English language' and 'not considered a quantity in Quinex'

*** the parser returns whether it likely succeeded or not

Table S15. Failure modes observed in the evaluation of the rule-based quantity parser, showing the total number of errors made in each category and the percentage share relative to the total number of errors (see Table S14).

Error type	Corpus 1 (unseen)	Corpus 1 (seen)	Corpus 2 (unseen)	Corpus 2 (seen)
Quantity rules not elaborate enough (sum of sub-types below)	13 (21.0%)	7 (22.6%)	116 (42.0%)	67 (39.0%)
- Segmentation in individual quantities, values, units, and/or modifiers wrong	2 (3.2%)	1 (3.2%)	44 (15.9%)	6 (3.5%)
- Unexpected quantity surface form (e.g., ‘$0.2 \leq x \leq 0.5$’)	1 (1.6%)	1 (3.2%)	21 (7.6%)	21 (12.2%)
- Cannot distinguish units from text to be ignored (e.g., Italy in ‘2% (Italy) and 4%’)	5 (8.1%)	5 (16.1%)	14 (5.1%)	14 (8.1%)
- Quantity is complex list (e.g., ‘(ppm):7.3(1H, d, J=5Hz), 7.2(1H, d, J=6Hz), ...’)	0 —	0 —	16 (5.8%)	16 (9.3%)
- Rules not elaborate enough in other respects (e.g., considering ‘an hour’ as 1 hour)	0 —	0 —	12 (4.3%)	2 (1.2%)
- Quantity describes an interval and not a list only apparent from context	0 —	0 —	5 (1.8%)	5 (2.9%)
- Imprecise quantity not in gazetteer (e.g., ‘hundreds of millions’)	5 (8.1%)	0 —	0 —	0 —
- Unit ellipsis resolution only for suffixed units (e.g., for ‘4 to 5 N’ but not ‘N 4 - 5’)	0 —	0 —	2 (0.7%)	2 (1.2%)
- Constants not considered (e.g., in ‘ambient pressure to 3 GPa’)	0 —	0 —	1 (0.4%)	1 (0.6%)
- Quantity modifier not in gazetteer (e.g., ‘non-’ in ‘non-zero’)	0 —	0 —	1 (0.4%)	0 —
QUDT unit linking not elaborate enough (sum of sub-types below)	32 (51.6%)	7 (22.6%)	72 (26.1%)	30 (17.4%)
- Disambiguation based on hard-coded priorities without considering context	1 (1.6%)	1 (3.2%)	16 (5.8%)	17 (9.9%)
- Unit in gazetteers but not with given surface form (e.g., ‘gigabit’ but not ‘Gbit’)	6 (9.7%)	0 —	26 (9.4%)	0 —
- Rules not elaborate enough in other respects (e.g., unexpected dash in ‘μ-Ωcm’)	19 (30.6%)	0 —	6 (2.2%)	1 (0.6%)
- Cannot deal with unit modifiers (e.g., H2 in ‘kgH2’ or consec. in ‘consecutive days’)	6 (9.7%)	6 (19.4%)	4 (1.4%)	4 (2.3%)
- Unit not in gazetteers (not in QUDT and not manually added)	0 —	0 —	9 (3.3%)	1 (0.6%)
- Unit in gazetteers but not with given prefix (e.g., Oe exists but not kOe)	0 —	0 —	7 (2.5%)	3 (1.7%)
- Cannot link to QUDT if non-physical units present (e.g., molecule in ‘molecule/s’)	0 —	0 —	2 (0.7%)	2 (1.2%)
- Unit only apparent from context (e.g., unit only stated once at start of paragraph)	0 —	0 —	2 (0.7%)	2 (1.2%)
OCR/PDF parsing error (e.g., ‘1mjs’ instead of ‘1 m/s’)	16 (25.8%)	16 (51.6%)	35 (12.7%)	35 (20.3%)
Non-English language (e.g., ‘1 bis 2 Prozent’)	0 (0.0%)	0 (0.0%)	32 (11.6%)	32 (18.6%)
String to number conversion not elaborate enough (e.g., ‘1x107’ to 107 vs. 1e7)	0 (0.0%)	0 (0.0%)	15 (5.4%)	2 (1.2%)
Not considered a quantity in Quinex (e.g., dates or time of day)	1 (1.6%)	1 (3.2%)	6 (2.2%)	6 (3.5%)

S4 Curation frontend and annotation format

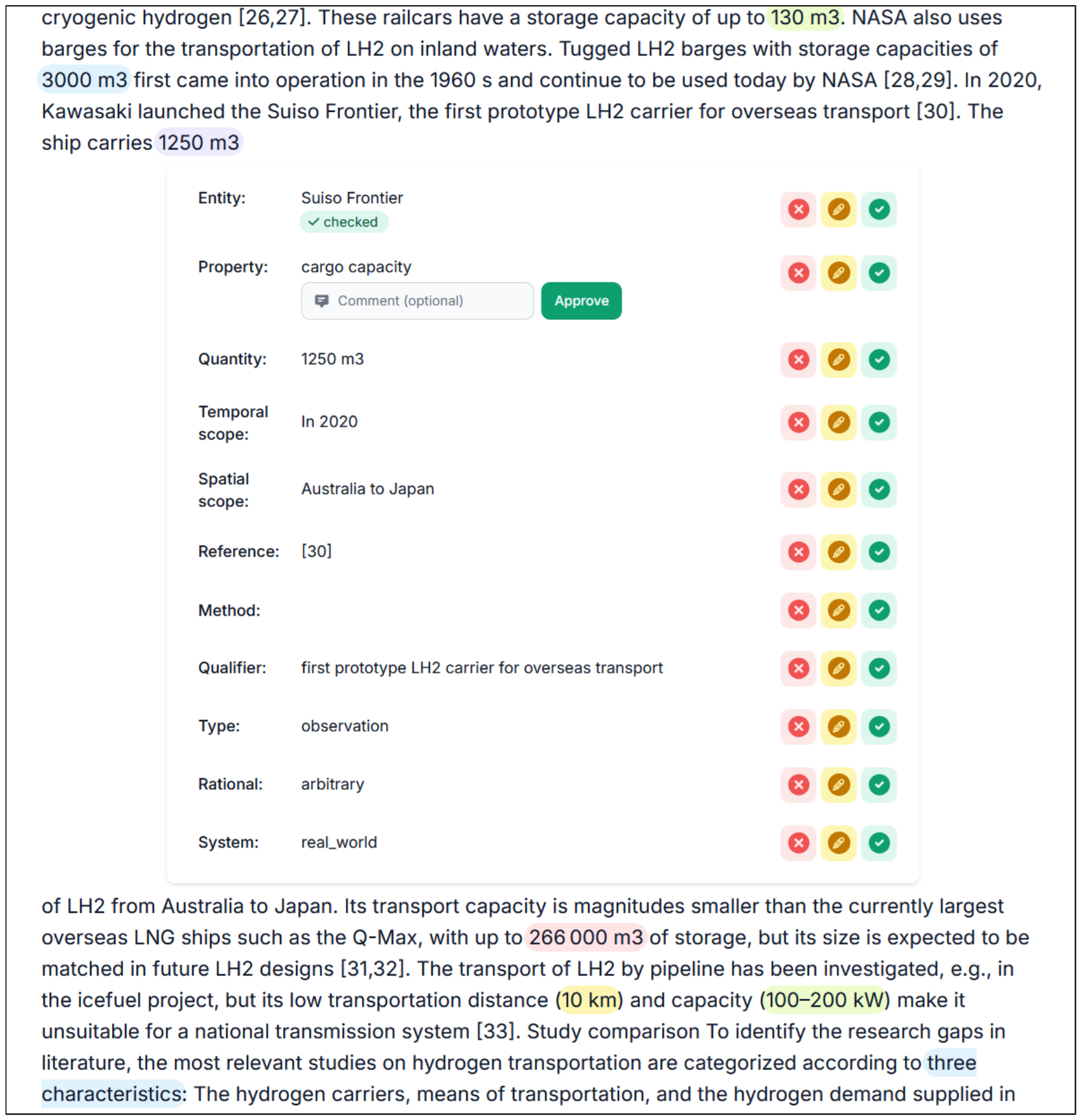

Figure S4. Frontend for visualization and curation of Quinex predictions.

a)

```
{
    "id": 52633,
    "source": "Suldal (en:178082)",
    "text": "The finished hydroelectric complex has a total capacity of approximately 🍏2,100
        megawatts🍏 and produces about 🍏4.8 terawatt-hours🍏 yearly (Figure S2).",
    "correct": null
}
```

b)

```
{
    "id": 28066,
    "source": "Colongra Power Station (en:25606789)",
    "text": "🌶️Colongra Gas Generation Plant (also known as Colongra Power Station)🌶️ is a
           🍏667 MW🍏 gas-fired 🍊power🍊 station located in 📍Colongra, New South Wales,
           Australia📍 and is the largest gas-fired power station in New South Wales.",
    "implicit_property": "installed capacity",
    "curation": {
        "quantity_entity_property_correct": true,
        "spatio_temporal_scope_correct": true,
        "qualifiers_correct": true
    }
}
```

Figure S5. The annotation format uses colorful emojis to simplify dataset curation for **(a)** quantity span identification and **(b)** measurement context extraction. Figure adapted from Göpfert et al. (2025).[32] Copyright 2025, Elsevier.

S5 Question templates

Table S16 shows the selected set of question templates. Section S5.1 & S5.2 list all variants tested when asking for the measured property first and the entity first, respectively.

Table S16. Best set of question templates testing a permutation of entity and property question templates as well as different entity fallback questions (see Section S3.2.2). The question templates for qualifier extraction have not been optimized.

Concept	Question template
Property	Default: "Which property or quality is characterized by {*quantity_span*}?"
Entity	Default: "Which entity's {*property_span*} {*is_or_are*} characterized by {*quantity_span*}?"<br>Fallback: "Which entity does {*quantity_span*} characterize?"
Temporal Scope	Default: "For which point in time is the statement true that {*property_span*} of {*entity_span*} {*is_or_are*} {*quantity_span*}?"<br>Fallback: "For which point in time is the statement true that {*entity_or_property_span*} {*is_or_are*} {*quantity_span*}?"
Spatial Scope	Default: "For which location is the statement true that {*property_span*} of {*entity_span*} {*is_or_are*} {*quantity_span*}?"<br>Fallback: "For which location is the statement true that {*entity_or_property_span*} {*is_or_are*} {*quantity_span*}?"
References	Default: "According to whom or which reference is the statement true that {*property_span*} of {*entity_span*} {*is_or_are*} {*quantity_span*}?"<br>Fallback: "According to whom or which reference is the statement true that {*entity_or_property_span*} {*is_or_are*} {*quantity_span*}?"
Methods	Default: "What methods and instruments were used in determining that {*property_span*} of {*entity_span*} {*is_or_are*} {*quantity_span*}?"<br>Fallback: "What methods and instruments were used in determining that {*entity_or_property_span*} {*is_or_are*} {*quantity_span*}?"
Other Qualifiers	Default: "Under which constraints is the statement true that {*property_span*} of {*entity_span*} {*is_or_are*} {*quantity_span*}?"<br>Fallback: "Under which constraints is the statement true that {*entity_or_property_span*} {*is_or_are*} {*quantity_span*}?"

S5.1 Tested question templates (property-first)

The following is a list of all question templates that were tested when asking for the measured property before the entity.

Property questions:

- "Property: "
- "Which property is described by {*quantity_span*}?"
- "What property is described by {*quantity_span*}?"
- "Which property has a value of {*quantity_span*}?"
- "Which property is {*quantity_span*}?"
- "Which attribute is {*quantity_span*}?"
- "Which quality is {*quantity_span*}?"
- "Which characteristic is {*quantity_span*}?"
- "Which parameter is {*quantity_span*}?"
- "Which property/attribute/parameter is {*quantity_span*}?"
- "Which property, attribute, or parameter is {*quantity_span*}?"
- "Which property, attribute, quality, or parameter is {*quantity_span*}?"
- "Which property (attribute, quality, characteristic or parameter) is {*quantity_span*}?"
- "Which attribute or quality is {*quantity_span*}?"
- "Which property or quality is {*quantity_span*}?"
- "Which property or attribute is {*quantity_span*}?"
- "Which property or characteristic is {*quantity_span*}?"
- "Which property or parameter is {*quantity_span*}?"
- "Which property, attribute, or parameter has a value of {*quantity_span*}?"
- "Which property is characterized by {*quantity_span*}?"
- "Which attribute is characterized by {*quantity_span*}?"
- "Which quality is characterized by {*quantity_span*}?"
- "Which parameter is characterized by {*quantity_span*}?"
- "Which property/attribute/parameter is characterized by {*quantity_span*}?"
- "Which property, attribute, or parameter is characterized by {*quantity_span*}?"
- "Which property, attribute, quality, or parameter is characterized by {*quantity_span*}?"
- "Which property (attribute, quality, or parameter) is characterized by {*quantity_span*}?"
- "Which attribute or quality is characterized by {*quantity_span*}?"

- "Which property or quality is characterized by {*quantity_span*}?"
- "Which property or attribute is characterized by {*quantity_span*}?"
- "Which property or parameter is characterized by {*quantity_span*}?"
- "Which property, attribute, or parameter is characterized by a value of {*quantity_span*}?"
- "Which property does {*quantity_span*} characterize?"
- "Which property is quantifed by {*quantity_span*}?"
- "Which property does {*quantity_span*} quantify?"
- "Which property does {*quantity_span*} relate to?"
- "Which property is being described by {*quantity_span*}?"
- "Which property is described by the highlighted quantity?"
- "Which property is described by the highlighted quantity {*quantity_span*}?"

Entity questions:

- "Entity: "
- "Which entity has a {*property_span*} of {*quantity_span*}?"
- "What entity has a {*property_span*} of {*quantity_span*}?"
- "Which entity (thing, person or process) has a {*property_span*} of {*quantity_span*}?"
- "Which entity or process has a {*property_span*} of {*quantity_span*}?"
- "Which object, person or process has a {*property_span*} of {*quantity_span*}?"
- "Which entity of {*property_span*} does {*quantity_span*} describe?"
- "What entity of {*property_span*} does {*quantity_span*} describe?"
- "Which entity's {*property_span*} is {*quantity_span*}?"
- "Which entity's {*property_span*} is characterized by {*quantity_span*}?"
- "Which entity's {*property_span*} is quantified by {*quantity_span*}?"
- "Which entity's {*property_span*} is described by {*quantity_span*}?"
- "Which entity's {*property_span*} is being described by {*quantity_span*}?"
- "The {*property_span*} of which entity is {*quantity_span*}?"
- "The {*property_span*} of which entity is characterized by {*quantity_span*}?"
- "The {*property_span*} of which entity (thing, person or process) is characterized by {*quantity_span*}?"
- "The {*property_span*} of which entity or process is characterized by {*quantity_span*}?"
- "The {*property_span*} of which object, person or process is characterized by {*quantity_span*}?"

Fallback entity questions:

- "Which entity does {*quantity_span*} describe?"
- "Which entity does {*quantity_span*} characterize?"
- "Which entity does {*quantity_span*} quantify?"
- "Which entity does {*quantity_span*} relate to?"
- "Which entity is described by {*quantity_span*}?"
- "Which entity is characterized by {*quantity_span*}?"
- "Which entity has a property that is {*quantity_span*}?"
- "Which entity has a property or quality that is {*quantity_span*}?"

S5.2 Tested question templates (entity-first)

The following is a list of all question templates that were tested when asking for the measured entity before the property.

Entity questions:

- "Entity: "
- "Which entity is characterized by {*quantity_span*}?"
- "Which entity is being characterized by {*quantity_span*}?"
- "Which entity (thing, person or process) is characterized by {*quantity_span*}?"
- "Which entity, person or process is characterized by {*quantity_span*}?"
- "Which entity or process is characterized by {*quantity_span*}?"
- "What entity or process is characterized by {*quantity_span*}?"
- "Which object, person or process is characterized by {*quantity_span*}?"
- "What object, person or process is characterized by {*quantity_span*}?"
- "Which item, person or process is characterized by {*quantity_span*}?"
- "Which subject, person or process is characterized by {*quantity_span*}?"
- "Which thing, person or process is characterized by {*quantity_span*}?"
- "Which entity has a property that is {*quantity_span*}?"
- "Which entity does {*quantity_span*} describe?"
- "What entity does {*quantity_span*} describe?"

- "Which entity does {*quantity_span*} quantify?"
- "Which entity does {*quantity_span*} relate to?"
- "Which entity is described by {*quantity_span*}?"
- "Which entity is being described by {*quantity_span*}?"
- "Which entity is described by the highlighted quantity?"
- "Which entity is described by the highlighted quantity {*quantity_span*}?"

Property questions:

- "Property:"
- "Which property of {*entity_span*} is {*quantity_span*}?"
- "What property of {*entity_span*} is {*quantity_span*}?"
- "Which attribute of {*entity_span*} is {*quantity_span*}?"
- "Which quality of {*entity_span*} is {*quantity_span*}?"
- "Which characteristic of {*entity_span*} is {*quantity_span*}?"
- "Which parameter of {*entity_span*} is {*quantity_span*}?"
- "Which property/attribute/parameter is {*quantity_span*}?"
- "Which property, attribute, or parameter is {*quantity_span*}?"
- "Which property of {*entity_span*} does {*quantity_span*} quantify?"
- "Which property of {*entity_span*} does {*quantity_span*} relate to?"
- "Which property of {*entity_span*} does {*quantity_span*} characterize?"
- "Which property of {*entity_span*} is described by {*quantity_span*}?"
- "Which property of {*entity_span*} is being described by {*quantity_span*}?"
- "Which property of {*entity_span*} is characterized by {*quantity_span*}?"
- "Which property of {*entity_span*} is being characterized by {*quantity_span*}?"
- "Which property of the highlighted entity is described by the highlighted quantity?"
- "Which property of the highlighted entity {*entity_span*} is described by the highlighted quantity {*quantity_span*}?"

S6 Tested enclosures

Below is a list of all the enclosures that have been tested. In addition, not enclosing previous predictions was tested. Note that, although the enclosures are grouped into a single category, some enclosures overlap in use (e.g., Markdown supports HTML, '$' is used for math environment in LaTeX and also many Markdown syntaxes). Also, some characters are not in the vocabulary of the T5 tokenizer.

- **Markdown**: **...**, ~...~, [...], __...__, '...'
- **Arrows and pointer**: =>...<=, ->...<-, -->...<--, ▌☞...☜▌, ▶...◀
- **LaTeX**: $...$, \(...\), \quantity{...}, \property{...}
- **MediaWiki markup**: [[...]]
- **Emojis**: 🍏...🍏 , 🌶...🌶, 👈...👉, ➡️...⬅️, ❌...❌,
- **HTML/XML tags**: ..., ..., ..., ..., ..., ..., <b class="QUANTITY">..., <b class="PROPERTY">..., <i>...</i>, <u>...</u>, ..., ..., <mark>...</mark>, <mark class="QUANTITY">...</mark>, <mark class="PROPERTY">...</mark>
- **Template engines**: {...}, {{...}}
- **Quotation and question marks**: «...», ¿...?
- **ANSI escape codes**: \033[91m...\033[0m
- **Other**: @@@...@@@, @...@, ==...==, ^^...^^, |...|, ||...||, //...//, #...#, ##...##, <...>, <<...>>, •...•, —...—, ['...'], !(...)!, !!!...!!!, :::...:::

S7 Search queries for application experiments

The following Scopus search queries were used in the application experiments:

- Levelized cost of electricity: `( TITLE-ABS-KEY ( ( "lcoe" OR "lcoes" OR "levelised cost of electricity" OR "levelized cost of electricity" ) AND ( "kWh" OR "kW h" OR "MWh" OR "MW h" ) ) AND LANGUAGE ( english ) )`, executed on 2024-10-23.

- Maximal oxygen uptake: `TITLE-ABS-KEY (("vo2 max" OR "VO2(max)" OR "maximal oxygen consumption" OR "maximal oxygen uptake" OR "maximal aerobic capacity") AND "ml" AND "kg" and "min") AND (LIMIT-TO (LANGUAGE , "English")) AND (LIMIT-TO (SRCTYPE , "j")) AND (LIMIT-TO (DOCTYPE , "ar") OR LIMIT-TO (DOCTYPE , "re"`

`) ) AND ( LIMIT-TO ( SUBJAREA , "MEDI" ) OR LIMIT-TO ( SUBJAREA , "HEAL" ) OR LIMIT-TO ( SUBJAREA , "BIOC" ) )`, executed on 2025-07-29.

- Earthquake moment magnitudes: `ABS ( earthquake AND ( "moment magnitude" OR "surface wave magnitude" OR "body wave magnitude" OR "local magnitude" ) AND ( 1 OR 2 OR 3 OR 4 OR 5 OR 6 OR 7 OR 8 OR 9 ) ) AND ( LIMIT-TO ( LANGUAGE , "English" ) ) )`, executed on 2025-07-30.

- Perovskite bandgaps: `TITLE-ABS-KEY ( ( {eV} OR {(ev)} OR "electronvolt" OR "electron-volt" OR "electron volt" ) AND ( "bandgap" OR "band-gap" OR "band gap" OR "bandedge" OR "band-edge" OR "band edge" ) AND ( ( "methylammonium lead iodide" OR "methylammonium lead triiodide" OR {ch3nh3pbi3} OR {mapbi3} OR ...` (continuing with the remaining material synonyms in Sipilä et al.[33] S2), executed on 2025-07-29.

REFERENCES

1. Ray, S. Quantities, Units, Dimensions and Types, 10.25504/FAIRsharing.d3pqw7 (2011).
2. Rijgersberg, H., Wigham, M. & Top, J. L. How semantics can improve engineering processes: A case of units of measure and quantities. *Adv. Eng. Informatics* **25**, 276–287, 10.1016/j.aei.2010.07.008 (2011).
3. Almasian, S., Kazakova, V., Göldner, P. & Gertz, M. CQE: A Comprehensive Quantity Extractor. In Bouamor, H., Pino, J. & Bali, K. (eds.) *Proceedings of the 2023 Conference on Empirical Methods in Natural Language Processing*, 12845–12859, 10.18653/v1/2023.emnlp-main.793 (Association for Computational Linguistics, Singapore, 2023).
4. quantulum3 (2016–2025). URL: https://github.com/nielstron/quantulum3.
5. Vrandečić, D. & Krötzsch, M. Wikidata: a free collaborative knowledgebase. *Commun. ACM* **57**, 78–85, 10.1145/2629489 (2014).
6. semchunk (2023–2025). URL: https://github.com/isaacus-dev/semchunk.
7. Davletov, A., Gordeev, D., Arefyev, N. & Davletov, E. LIORI at SemEval-2021 Task 8: Ask Transformer for measurements. In *Proceedings of the 15th International Workshop on Semantic Evaluation (SemEval-2021)*, 1249–1254, 10.18653/v1/2021.semeval-1.178 (Association for Computational Linguistics, Online, 2021).
8. Harper, C. *et al.* SemEval-2021 Task 8: MeasEval – Extracting Counts and Measurements and their Related Contexts. In *Proceedings of the 15th International Workshop on Semantic Evaluation (SemEval-2021)*, 306–316, 10.18653/v1/2021.semeval-1.38 (Association for Computational Linguistics, Online, 2021).
9. Li, Y., Martschat, S. & Ponzetto, S. P. Multi-Source (Pre-)Training for Cross-Domain Measurement, Unit and Context Extraction. In Demner-fushman, D., Ananiadou, S. & Cohen, K. (eds.) *The 22nd Workshop on Biomedical Natural Language Processing and BioNLP Shared Tasks*, 1–25, 10.18653/v1/2023.bionlp-1.1 (Association for Computational Linguistics, Toronto, Canada, 2023).
10. Foppiano, L., Romary, L., Ishii, M. & Tanifuji, M. Automatic Identification and Normalisation of Physical Measurements in Scientific Literature. In *Proceedings of the ACM Symposium on Document Engineering 2019*, 1–4, 10.1145/3342558.3345411 (ACM, Berlin Germany, 2019).
11. Bhattacharjee, B. *et al.* INDUS: Effective and efficient language models for scientific applications. In Dernoncourt, F., Preoţiuc-Pietro, D. & Shimorina, A. (eds.) *Proceedings of the 2024 Conference on Empirical Methods in Natural Language Processing: Industry Track*, 98–112, 10.18653/v1/2024.emnlp-industry.9 (Association for Computational Linguistics, Miami, Florida, US, 2024).
12. Gururangan, S. *et al.* Don't Stop Pretraining: Adapt Language Models to Domains and Tasks. In Jurafsky, D., Chai, J., Schluter, N. & Tetreault, J. (eds.) *Proceedings of the 58th Annual Meeting of the Association for Computational Linguistics*, 8342–8360, 10.18653/v1/2020.acl-main.740 (Association for Computational Linguistics, Online, 2020).
13. Webersinke, N., Kraus, M., Bingler, J. A. & Leippold, M. ClimateBert: A Pretrained Language Model for Climate-Related Text, 10.48550/arXiv.2110.12010 (2022).
14. Tinn, R. *et al.* Fine-tuning large neural language models for biomedical natural language processing. *Patterns* **4**, 100729, 10.1016/j.patter.2023.100729 (2023).
15. Liu, Y. *et al.* RoBERTa: A Robustly Optimized BERT Pretraining Approach, 10.48550/arXiv.1907.11692 (2019).
16. Conneau, A. *et al.* Unsupervised Cross-lingual Representation Learning at Scale. In Jurafsky, D., Chai, J., Schluter, N. & Tetreault, J. (eds.) *Proceedings of the 58th Annual Meeting of the Association for Computational Linguistics*, 8440–8451, 10.18653/v1/2020.acl-main.747 (Association for Computational Linguistics, Online, 2020).
17. Rob Srebrovic & Jay Yonamine. Leveraging the BERT algorithm for Patents with TensorFlow and BigQuery. Tech. Rep., Google (2020). URL: https://services.google.com/fh/files/blogs/bert_for_patents_white_paper.pdf.
18. Luca Foppiano & Pedro Ortiz Suarez. Material SciBERT (TPU): Improving language understanding in materials science (2022). URL: https://huggingface.co/lfoppiano/MatTPUSciBERT.
19. Schimanski, T. *et al.* Bridging the gap in ESG measurement: Using NLP to quantify environmental, social, and governance communication. *Finance Res. Lett.* **61**, 104979, 10.1016/j.frl.2024.104979 (2024).
20. Beltagy, I., Lo, K. & Cohan, A. SciBERT: A Pretrained Language Model for Scientific Text. In *Proceedings of the 2019 Conference on Empirical Methods in Natural Language Processing and the 9th International Joint Conference on Natural Language Processing (EMNLP-IJCNLP)*, 3613–3618, 10.18653/v1/D19-1371 (Association for Computational Linguistics, Hong Kong, China, 2019).

21. Sanh, V., Debut, L., Chaumond, J. & Wolf, T. DistilBERT, a distilled version of BERT: Smaller, faster, cheaper and lighter, 10.48550/arXiv.1910.01108 (2020). 1910.01108.

22. Devlin, J., Chang, M.-W., Lee, K. & Toutanova, K. BERT: Pre-training of Deep Bidirectional Transformers for Language Understanding. In *Proceedings of the 2019 Conference of the North American Chapter of the Association for Computational Linguistics: Human Language Technologies, Volume 1 (Long and Short Papers)*, 4171–4186, 10.18653/v1/N19-1423 (Association for Computational Linguistics, Minneapolis, Minnesota, 2019).

23. Alsentzer, E. *et al.* Publicly Available Clinical BERT Embeddings, 10.48550/arXiv.1904.03323 (2019). 1904.03323.

24. Sun, Z. *et al.* MobileBERT: A Compact Task-Agnostic BERT for Resource-Limited Devices. In Jurafsky, D., Chai, J., Schluter, N. & Tetreault, J. (eds.) *Proceedings of the 58th Annual Meeting of the Association for Computational Linguistics*, 2158–2170, 10.18653/v1/2020.acl-main.195 (Association for Computational Linguistics, Online, 2020).

25. Rohanian, O., Nouriborji, M., Kouchaki, S. & Clifton, D. A. On the effectiveness of compact biomedical transformers. *Bioinformatics* **39**, btad103, 10.1093/bioinformatics/btad103 (2023).

26. Jiao, X. *et al.* TinyBERT: Distilling BERT for Natural Language Understanding. In Cohn, T., He, Y. & Liu, Y. (eds.) *Findings of the Association for Computational Linguistics: EMNLP 2020*, 4163–4174, 10.18653/v1/2020.findings-emnlp.372 (Association for Computational Linguistics, Online, 2020).

27. Rohanian, O. *et al.* Lightweight transformers for clinical natural language processing. *Nat. Lang. Eng.* **30**, 887–914, 10.1017/S1351324923000542 (2024).

28. Yoon, W., Jackson, R., Ford, E., Poroshin, V. & Kang, J. Biomedical NER for the Enterprise with Distillated BERN2 and the Kazu Framework. In Li, Y. & Lazaridou, A. (eds.) *Proceedings of the 2022 Conference on Empirical Methods in Natural Language Processing: Industry Track*, 619–626, 10.18653/v1/2022.emnlp-industry.63 (Association for Computational Linguistics, Abu Dhabi, UAE, 2022).

29. Weischedel, R. *et al.* OntoNotes Release 5.0, 10.35111/XMHB-2B84 (2013).

30. Chung, H. W. *et al.* Scaling Instruction-Finetuned Language Models. *J. Mach. Learn. Res.* **25**, 1–53 (2024). URL: http://jmlr.org/papers/v25/23-0870.html.

31. grobid-quantities (2015–2024). Swh:1:dir:dbf9ee55889563779a09b16f9c451165ba62b6d7. URL: https://github.com/kermitt2/grobid-quantities.

32. Göpfert, J., Kuckertz, P., Weinand, J. M. & Stolten, D. Wiki-Quantities and Wiki-Measurements: Datasets of quantities and their measurement context from Wikipedia. *Sci. Data* **12**, 1277, 10.1038/s41597-025-05499-3 (2025).

33. Sipilä, M., Mehryary, F., Pyysalo, S., Ginter, F. & Todorović, M. Question Answering models for information extraction from perovskite materials science literature. *Commun. Mater.* **6**, 260, 10.1038/s43246-025-00979-w (2025).
